# Clinical potential and experimental validation of prognostic genes in hepatocellular carcinoma revealed by risk modeling utilizing single cell and transcriptome constructs

**DOI:** 10.3389/fimmu.2025.1541252

**Published:** 2025-04-04

**Authors:** Hang Deng, Xu Wang, Zi-Ang Jiang, Jian Xu, Yu Zhang, Yao Zhou, Jun Gong, Xiang-Yu Lu, Yi-Fu Hou, Hao Zhang

**Affiliations:** ^1^ Medical College, University of Electronic Science and Technology of China, Chengdu, China; ^2^ Department of Hepatobiliary Surgery, Sichuan Provincial People’s Hospital, University of Electronic Science and Technology of China, Chengdu, China; ^3^ Medical College, North Sichuan Medical College, Nanchong, China; ^4^ Department of Organ Translation Center, Sichuan Provincial People’s Hospital, University of Electronic Science and Technology of China, Chengdu, China

**Keywords:** hepatocellular carcinoma, single-cell sequencing analysis, combination algorithms, prognostic genes, drug sensitivity

## Abstract

**Background:**

Hepatocellular carcinoma (HCC) is the leading cause of tumor-related mortality worldwide. There is an urgent need for predictive biomarkers to guide treatment decisions. This study aimed to identify robust prognostic genes for HCC and to establish a theoretical foundation for clinical interventions.

**Methods:**

The HCC datasets were obtained from public databases and then differential expression analysis were used to obtain significant gene expression profiles. Subsequently, univariate Cox regression analysis and PH assumption test were performed, and a risk model was developed using an optimal algorithm from 101 combinations on the TCGA-LIHC dataset to pinpoint prognostic genes. Immune infiltration and drug sensitivity analyses were conducted to assess the impact of these genes and to explore potential chemotherapeutic agents for HCC. Additionally, single-cell analysis was employed to identify key cellular players and their interactions within the tumor microenvironment. Finally, reverse transcription-quantitative polymerase chain reaction (RT-qPCR) was utilized to validate the roles of these prognostic genes in HCC.

**Results:**

A total of eight prognostic genes were identified (MCM10, CEP55, KIF18A, ORC6, KIF23, CDC45, CDT1, and PLK4). The risk model, constructed based on these genes, was effective in predicting survival outcomes for HCC patients. CEP55 exhibited the strongest positive correlation with activated CD4 T cells. The top 10 drugs showed increased sensitivity in the low-risk group. B cells were identified as key cellular components with the highest interaction numbers and strengths with macrophages in both HCC and control groups. Prognostic genes were more highly expressed in the initial state of B cell differentiation. RT-qPCR confirmed significant upregulation of MCM10, KIF18A, CDC45, and PLK4 in HCC tissues (p< 0.05).

**Conclusion:**

This study successfully identified eight prognostic genes (MCM10, CEP55, KIF18A, ORC6, KIF23, CDC45, CDT1, and PLK4), which provided new directions for exploring the potential pathogenesis and clinical treatment research of HCC.

## 1 Introduction

Hepatocellular carcinoma (HCC) is the sixth most common malignant tumor globally and the third leading cause of cancer-related deaths (1). Hepatitis B, hepatitis C, and alcoholic and nonalcoholic fatty liver diseases are the most common etiologies of HCC (2). In China, the prevalence of HCC is particularly concerning, with a majority of patients presenting with advanced disease stages, often precluding surgical intervention, which is the preferred treatment modality for long-term survival and potential cure (3). Given the complexity of the etiologies and the insidious onset of HCC, a multidisciplinary approach is essential in treatment. Despite the availability of surgical options for resectable HCC, it is crucial to integrate the expertise of surgeons, medical oncologists, radiation oncologists, and interventional radiologists to optimize patient care. This collaborative strategy tailors individual treatment plans based on tumor staging, liver function, and performance status, considering both tumor and patient related factors, such as genetic mutations driving incidence and mortality rates (4). Despite the approval of new drugs and the application of immunotherapies, which have led to improved prognoses for advanced HCC patients (5), there is still a significant variation in overall survival rates among HCC patients (6). In addition to curative surgery, there are numerous local and systemic treatment options available and the therapeutic strategies require further optimization. The identification of novel prognostic biomarkers could offer valuable insights for tailoring treatment approaches and refining treatment protocols, which may subsequently improve the prognosis of HCC patients.

The rapid advancement of single-cell technologies has positioned single-cell transcriptomics as a key tool for uncovering cellular heterogeneity and complexity. Single-cell RNA sequencing (scRNA-seq) has provided valuable insights into cellular differentiation, tumor heterogeneity, and growth, establishing itself as a cutting-edge technology in biological research (7). By integrating single-cell transcriptomics with other omics data, including proteomics, metabolomics, and epigenetics, researchers can obtain a more comprehensive view of cellular functions. This multi-omics integration not only deepens our understanding of cellular states and dynamics but also provides new perspectives for exploring disease mechanisms, facilitating the discovery of biomarkers and identification of therapeutic targets (8). Zhang et al. (9) harnessed the power of public databases to amalgamate scRNA-seq data with bulk RNA sequencing data. By deploying an array of sophisticated bioinformatics methodologies, they successfully developed prognostic signatures, exemplified by the 8-gene macrophage-related risk signature, which demonstrated robust predictive power across several external validation cohorts. This work highlights the pivotal role of scRNA-seq in unveiling cellular heterogeneity, observing the dynamic changes in cellular states during disease progression, and exploring the individual differences in treatment response, which plays a significant role in the field.

With the advancement of bioinformatics technology, numerous prognostic signatures for HCC have been developed to assess patient prognosis risk, such as ferroptosis (10) and N6-methyladenosine (m6A) (11). However, improper use of machine learning methods and limitations of machine learning algorithms have significantly hindered the clinical application of these prognostic genes (12, 13). Current machine learning-based bioinformatic approaches for prognostic gene analysis exhibit certain limitations that may hinder their clinical translation. Yang et al. (14) developed a HCC prognostic model using the least absolute shrinkage and selection operator (LASSO) method combined with univariate Cox regression analysis. However, emerging evidence suggests that the predictive performance of LASSO-derived models may substantially deteriorate in external validation cohorts (15). These findings highlight the critical need to employ more robust machine learning algorithms to establish stable and clinically applicable prognostic models for HCC.

In this study, transcriptomics data from public databases were used to identify genes associated with the prognosis of HCC using multiple analysis algorithms. Subsequently, a prognostic model was constructed based on these prognostic genes and validated its reliability. Furthermore, the role of these prognostic genes in HCC was also deeply explored through analyses of the tumor immune microenvironment, the construction of regulatory networks, and predictions of drug sensitivity. Finally, the expression and distribution patterns of these genes in key cellular populations were analyzed in detail using single-cell analysis. These findings provide a solid foundation for a deeper understanding of the molecular mechanisms of HCC and the development of novel therapeutic strategies, potentially revolutionizing HCC treatment approaches and facilitating the realization of personalized medicine.

## 2 Materials and methods

### 2.1 Acquisition of data

The GSE149614 (platform: GPL24676) data for HCC were acquired from Gene Expression Omnibus (GEO) (https://www.ncbi.nlm.nih.gov/geo/), containing 10 HCC tumor samples and 8 adjacent tumor samples. The gene expression profiles, clinical information, and survival information of the TCGA-LIHC cohort were acquired from the University of California Santa Cruz (UCSC) Xena database (https://xena.ucsc.edu/) (accessed June 26, 2024). TCGA-LIHC dataset included 369 HCC samples (HCC group) along with 50 control samples (control group). After excluding patients without complete survival information, 363 HCC samples with survival information were retained for risk model construction. Furthermore, the ICGC-LIRI-JP cohort was acquired from the International Cancer Genome Consortium (ICGC) database (https://dcc.icgc.org/) (accessed June 26, 2024). The ICGC-LIRI-JP contained 231 HCC tumor samples with survival information were utilized for risk model validation. Lastly, the GSE76427 and GSE54236 datasets were downloaded from the GEO database for the expression validation of prognostic genes (accessed January 22, 2025). The GSE76427 dataset contained 115 HCC tumor tissue samples and 52 normal control tissue samples (platform: GPL10558), while the GSE54236 dataset included 81 HCC tumor tissue samples and 80 normal control tissue samples (platform: GPL6480).

### 2.2 Analyzing differential expression

As a further step to obtain genes associated with differences between HCC and control groups, the differentially expressed mRNAs (DE-mRNAs) in the TCGA-LIHC dataset were identified by the DESeq2 (v 1.34.0) package (16) (p< 0.05 and |log_2_ fold change (FC)| >10.10.1 2). Similarly, the DE-miRNAs and DE-lncRNAs were identified also by DESeq2 (v 1.34.0) package (p< 0.05 and |log_2_ FC| > 0.5). Lastly, ggplot2 (v 3.3.5) (17)was utilized for volcano plots, while the pheatmap package (v 1.0.12) (https://CRAN.R-project.org/package=pheatmap) was employed to create a heatmap, visualizing the top 20 upregulated and downregulated genes based on log_2_ FC values in HCC samples.

### 2.3 Enrichment analysis and identification of candidate genes

In the study, the biological functions for DE-mRNAs were analyzed for Gene Ontology (GO) and Kyoto Encyclopedia of Genes and Genomes (KEGG) enrichment (p adj< 0.05) by clusterProfiler (v 4.6.0) package (18), then the top 2 ranked data according to p adj values from smallest to largest in GO enrichment were visualized in this study. A protein-protein interaction (PPI) network for DE-mRNAs was constructed utilizing a search tool for the retrieval of interaction gene/proteins (STRING, https://string-db.org) (confidence level ≥ 0.9). Finally, Cytoscape (v 3.9.1) (19) software was utilized to visualize the network. Finally, the molecular complex detection (MCODE) plugin in Cytoscape (v 3.9.1) software was then utilized to select the highest scoring sub-network (K-core = 2, degree cutoff = 2, node score cutoff = 0.2, max depth = 100), afterwards candidate genes were obtained.

### 2.4 Construction of a risk model

In TCGA-LIHC, the univariate Cox regression analysis of candidate genes was performed utilizing the survival (v 3.5.3) package (20) (hazard ratio (HR)≠1 and p value< 0.05), and the genes which passed the proportional hazard (PH) assumption test (p > 0.05) were defined as candidate prognostic genes. Subsequently, a consensus prediction model based on candidate prognostic genes was constructed by means of 10 machine learning algorithms, including LASSO, random survival forests (RSF), elastic networks (Enet), stepwise Cox, Ridge, Cox boost, partial least squares Cox regression (plsRcox), supervised principal components (SuperPC), generalized augmented regression models (GBM), and support vector machine-recursive feature elimination (SVM-RFE). The 101 different combinations of all algorithms were fitted in TCGA-LIHC and ICGC-LIRI-JP and the concordance index (C-index) of each combination was calculated. The higher the C-index, the stronger the predictive ability of the model. The model with the highest C-index was selected as the best model for obtaining prognostic genes.

Then prognostic genes were utilized for constructing the risk model, the formulaic representation was as follows:


$$\text{Riskscore}=\sum_{\text{i}=1}^{\text{n}}\text{coef}\left({\text{gene}}_{\text{i}}\right)*\text{expr}\left({\text{gene}}_{\text{i}}\right)$$

Where “risk score” represents the risk score, “coef” signifies the risk coefficients attributed to each specific gene, and “expr” represents the expression of the respective genes. Then, the risk model was evaluated by plotting the receiver operating characteristic (ROC) curves with the use of survivalROC (v 1.0.3) package (21) for the reliability of model. According to risk scores of prognostic genes in TCGA-LIHC, 363 HCC samples with survival information were divided into high- and low-risk groups (median score). Then, between two risk groups, the survminer (v 0.4.9) package (22) was utilized to generate Kaplan-Meier (KM) survival curves, and the log-rank test was utilized to analyze differences in survival between both groups (p< 0.05). In addition, after calculating the risk scores of the 231 HCC samples in ICGC-LIRI-JP, they were also divided into high- and low-risk groups based on the median risk score. After that, the generalizability of the risk model was confirmed by ROC curves, KM curves, risk curves, and prognostic gene expression heat maps. Furthermore, the model we constructed in this study was compared with previous models in the published literature (23–25), and the accuracy of the model constructed in the study was further compared by ROC analysis, KM survival curves, and C-index (p< 0.05).

### 2.5 Correlation of risk score with clinical characteristics

Firstly, a Wilcoxon test was utilized to compare difference between clinical characteristics with risk scores in TCGA-LIHC and visualized the results in a violin plot (p< 0.05). Secondly, the percentage of different clinical characteristics among HCC patients in two risk groups were showed in bar graphs. Finally, the chi-square tests were utilized to explore the distribution of different clinical characteristics between two risk groups and visualized the results in a heat map.

### 2.6 Gene set enrichment analysis and gene set variation analysis

In TCGA-LIHC, firstly, the HCC samples were analyzed the differences by DESeq2 (v 1.34.0) package and ranked the results based on the log_2_FC value of DE-mRNAs from largest to smallest. Additionally, the clusterProfiler (v 4.6.0) was used to perform the GSEA based on the “c2.cp.kegg.v2024.1.Hs.symbols” from the molecular signature database (MSigDB) (p adj< 0.05) (https://www.gsea-msigdb.org/gsea/msigdb). Following this, the GSEA results according to the p adj from smallest to largest were ranked. Then in the TCGA-LIHC, based on the “c5.go.v2023.1.Hs.symbols.gmt” from the MSigDB, the GSVA (v 1.42.0) package (26) and limma (v 3.50.1) package (27) were utilized to calculate GSVA scores for each pathway and to compare pathways that differ between two risk groups (|log_2_FC| > 0.5, p< 0.05), respectively. Finally, the top 10 regulated pathways were ranked according to the |log_2_FC| from smallest to largest and visualized in the end.

### 2.7 Immune microenvironment analysis

Then single-sample GSEA (ssGSEA) algorithm of GSVA (v 1.42.0) package was utilized to estimate the scores of 28 immune cells (28) in two risk groups and visualized in a heat map by pheatmap (v 1.0.12) package (29). Furthermore, an analysis of the difference in immune cell infiltration between two risk groups was conducted utilizing the Wilcoxon test (p< 0.05), with the results visualized in a box plot. After that, the correlations among differential immune cells were analyzed by psych (v 2.3.9) package (30) (|correlation coefficient (cor)| > 0.3, p< 0.05). So as to further gain insight into the potential relationships between differential immune cells and prognostic genes, the Spearman correlation analysis was conducted utilizing the ggcor (v 0.9.8.1) package (31) (|cor| > 0.3, p< 0.05).

Besides, a set of 24 immune checkpoints was compiled from previously published literature (32), and the expression of these checkpoints between two risk groups were analyzed (p< 0.05). Meanwhile, so as to explore the correlation between differential immune checkpoints and risk score, Spearman correlation analysis was performed in TCGA-LIHC (|cor| > 0.3, p< 0.05).

### 2.8 Analysis of mutated landscapes and ESTIMATE

To investigate the genetic differences between two risk groups in TCGA-LIHC, the maftools (v 2.10.5) package (33) of the R (v 4.2) language (34) was employed to analyze two cohorts of patients with mutation data. Additionally, the top 20 frequency mutated genes were visualized in a waterfall plot. Furthermore, in the TCGA-LIHC, the estimate (v 1.0.13) package (35) was utilized to calculate the StromalScore, ImmuneScore, and EstimateScore of the samples and the differences in these scores were compared (p< 0.001).

### 2.9 Regulatory networks construction

So as to search for miRNAs that potentially regulate prognostic genes, the starbase database (http://starbase.sysu.edu.cn/) was utilized as a predictor of miRNAs for prognostic genes. After that, the predicted miRNAs (Pre-miRNAs) and DE-miRNAs were taken to intersection, then the intersecting miRNAs in the opposite expression direction of prognostic genes regulation were selected and regarded as key miRNAs. Similarly, the mirnet database (https://www.mirnet.ca/) was utilized as a predictor of lncRNAs for key miRNAs. Following that, the predicted lncRNAs (Pre-lncRNAs) and DE-lncRNAs were taken to the intersection, then the intersecting lncRNAs in the opposite expression direction of key miRNAs regulation were selected and regarded as key lncRNAs. At last, the key lncRNAs-key miRNAs-prognostic genes regulatory network was visualized by Cytoscape (v 3.9.1) software.

Meanwhile, the upstream transcription factors (TFs) for prognostic genes were predicted via the JASPAR database (https://jaspar.elixir.no/) of the NetworkAnalyst platform (https://www.networkanalyst.ca/). At last, the TFs-prognostic genes regulatory network was mapped in the study.

### 2.10 Chemotherapeutic drug sensitivity analysis

The oncoPredict (v 0.1) (36) was utilized to obtain half maximal inhibitory concentration (IC_50_) values for HCC samples in TCGA-LIHC based on 198 chemotherapeutic drugs from genomics of drug sensitivity in cancer (GDSC) dataset (https://www.cancerrxgene.org/) and the IC_50_ between two risk groups for each drug was compared by Wilcoxon test (p< 0.05). Lastly, the results of top 10 drugs between two groups were ranged from smallest to largest based on p value and shown in the box plots.

### 2.11 Processing of the scRNA-seq data

In the GSE149614 dataset, the Seurat (v 5.0.1) package (37) was utilized for quality control and unsupervised cluster analysis. Firstly, cells with less than 200 genes and less than 3 cells covered with genes, the number of genes in each cell ≥ 8000, count in each cell ≥ 50000, and cells with greater than 10% of mitochondrial genes were filtered out. Furthermore, the vst method was utilized to obtain genes with relatively high coefficients of variation between cells, namely highly variable genes. Similarly, the JackStrawPlot function was employed to plot the scree plot to show the contribution of the top-ranked principal components (PCs) to cellular variation. After completing the principal components analysis (PCA) dimensionality reduction, the FindNeighbors and FindClusters functions (resolution = 0.1) were utilized to cluster the cells utilizing the uniform manifold approximation and projection (UMAP) method. After that, cell types were annotated utilizing the CellMarker dataset (http://117.50.127.228/CellMarker/) and previous literature (38), and visualized the expression of the marker genes in each cell type by means of violin plots. In addition, the ReactomeGSA (v 1.12.0) package (39) was utilized to analyze the pathways function of annotated cells, and the relationship between signaling and metabolic molecules was documented by Reactome database (https://reactome.org/), then the top 15 pathways with the greatest differences between different cell types were illustrated in a heat map.

### 2.12 Identification of key cells

In order to investigate the differential expression of prognostic genes among annotated cells between two groups in TCGA-LIHC, the Wilcoxon test was employed (p< 0.05). These cells were then defined as key cells for further analysis as they showed differential expression of prognostic genes in the HCC and control groups.

### 2.13 Analysis of intercellular interactions and cell trajectory

Intercellular communication was determined by the CellChat (v 1.6.1) package (40). The thicker the connection line between ligand and receptor reflected stronger potential interactions between cells. And the results of intercellular ligand-receptor interaction between key cells and other annotated cells were also shown in this study.

For studying the mechanism of key cells within other annotated cells, the differentiation of key cells was simulated utilizing the monocle (v 2.26.0) package (41) based on highly variable genes. In addition, the expression results of prognostic genes in different differentiation states of key cells were also shown in the end.

### 2.14 Dataset expression validation and reverse transcription-quantitative polymerase chain reaction

Finally, the Wilcoxon function from the “rstatix” package (v 0.7.2)(https://CRAN.R-project.org/package=rstatix) was used to analyze the expression differences of prognostic genes between HCC and control samples in the TCGA-LIHC, GSE76427, and GSE54236 datasets, and the results were displayed in boxplots. RNA was extracted from 10 samples using the TRizol kit, with 1-5 being adjacent tumor samples and 6-10 being HCC tumor samples, which were collected from the Sichuan Provincial People’s Hospital, University of Electronic Science and Technology of China. The study had been approved via the Ethics Committee of the Sichuan Provincial People’s Hospital, University of Electronic Science and Technology of China (Ethics Review [Res.] No. 192, 2023). All experimental steps for total RNA extraction were performed according to the instructions. 1 μL of extracted RNA was tested for concentration using a NanoPhotometer N50 and the purity/concentration was recorded to calculate the amount of RNA for subsequent reverse transcription steps. Subsequently, RNA was reverse transcribed into cDNA by SureScript-First-strand-cDNA-synthesis-kit (G3333-50, Servicebio) according to the instructions. cDNA was then diluted 5-20 times with ddH2O (without RNase/ARase), and 3 μL of cDNA, 5 μL of SweScript RT I Enzyme Mix (NO. 11904018, ThermoFisher), 1 μL forward primer (10 µM) and 1 μL reverse primer (10 µM). In addition, 40 cycles of reaction were performed using the CFX96 real-time quantitative PCR instrument (BIO-RAD), and the procedure information was shown in **Supplementary Table S1**. The primer sequences were shown in **Supplementary Table S2**, and GAPDH was used as a reference gene to determine the relative gene expression level by the 2^-ΔΔCT^ method.

### 2.15 Statistical analysis

R (v 4.2) language was utilized for all statistical analyses. Wilcoxon test was used to compare the differences between two groups. The value of p< 0.05 was considered statistically significant.

## 3 Results

### 3.1 Enrichment and PPI network analysis in 890 DE-mRNAs

**Figure 1:**
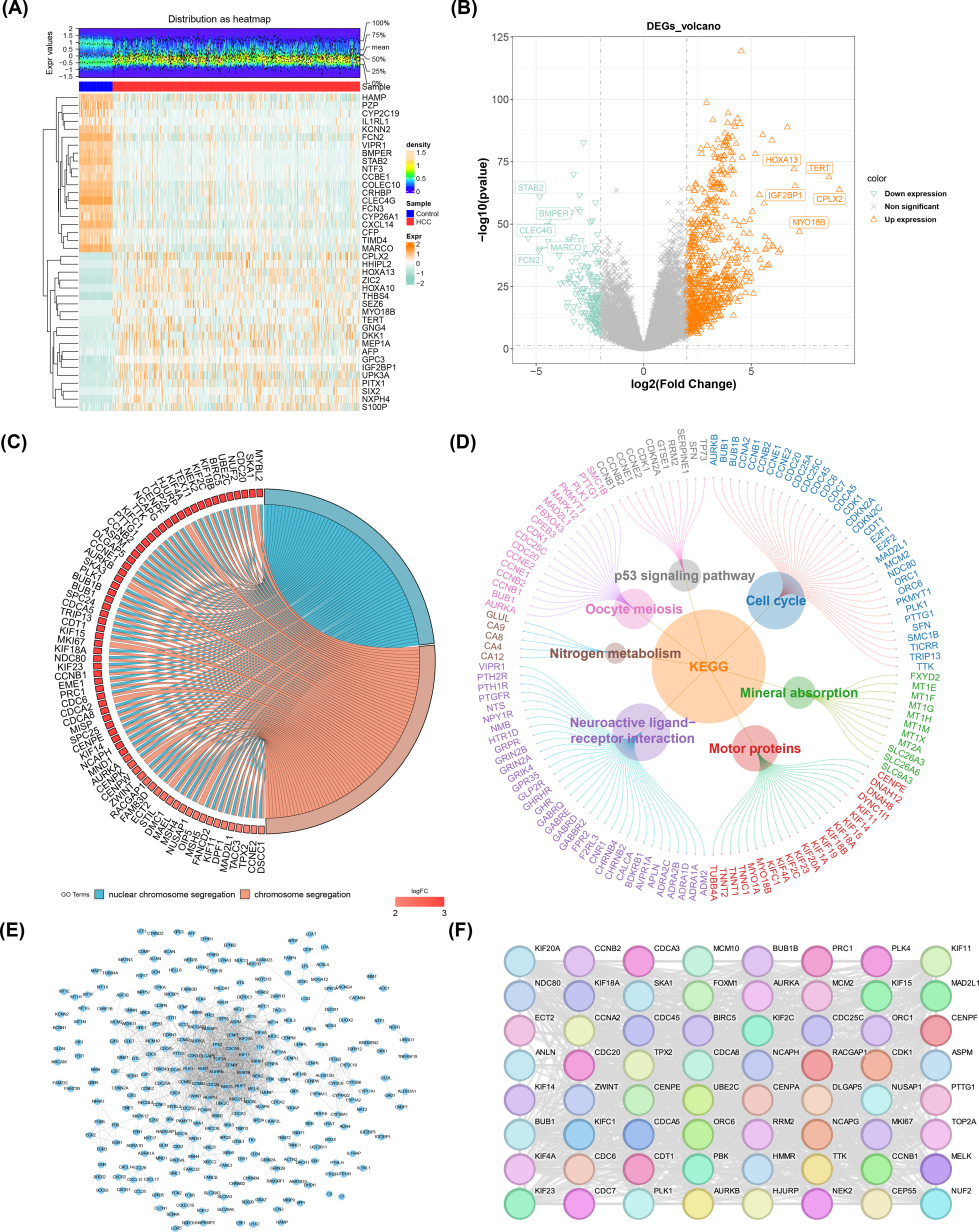
DE-mRNA identification, enrichment and PPI network analysis. **(A)** Heatmap of the top 20 upregulated and downregulated genes expression. The uppermost plot represented the differential mRNA expression density distribution, showing lines for the five percentiles and the average value; the lower plot, each square represented a sample, with orange indicating high expression and green indicating low expression. **(B)** Volcano map of 890 DE-mRNAs from TCGA-LIHC. **(C)** Gene Ontology (GO) enrichment analysis. **(D)** KEGG enrichment analysis. **(E)** PPI Network Diagram of Differentially Expressed Genes (DEGs). **(F)** Core candidate gene relationship network.

A total of 890 DE-mRNAs were obtained in TCGA-LIHC (|log_2_FC| > 2, p< 0.05) (n_up_ = 720, n_down_ = 170 in HCC group) (**Figures 1A, B**). According to the result of GO analysis, a sum of 298 biological processes (BPs), 34 cellular components (CCs), and 32 molecular functions (MFs) were enriched by DE-mRNAs. And the top 2 results of GO enrichment were visualized in circle diagram, including nuclear chromosome segregation and chromosome segregation (**Figure 1C**). In the BP assessment, they were mostly engaged in nuclear chromosome segregation. And mainly enriched in CC of chromosome, centromeric region. In addition, they were most enriched in MF of microtubule binding (**Supplementary Table S3**). Besides, a sum of 7 KEGG pathways were enriched and mainly enriched in cell cycle and neuroactive ligand-receptor interaction (p adj< 0.05) (**Figure 1D**; **Supplementary Table S4**). After that, a sum of 888 PPI networks of DEGs were obtained in the database (2 genes were not found to have corresponding proteins). And after removing the isolated nodes, a sum of 290 nodes and 1,263 sides were shown in a PPI network diagram (**Figure 1E**). Afterwards, a sum of 64 candidate genes were obtained in the end (**Figure 1F**; **Supplementary Table S5**). The results indicated that the 64 candidate genes had a strong association with HCC and were the basis for subsequent screening of prognostic genes.

### 3.2 Prognostic risk model was construct in TCGA-LIHC

**Figure 2:**
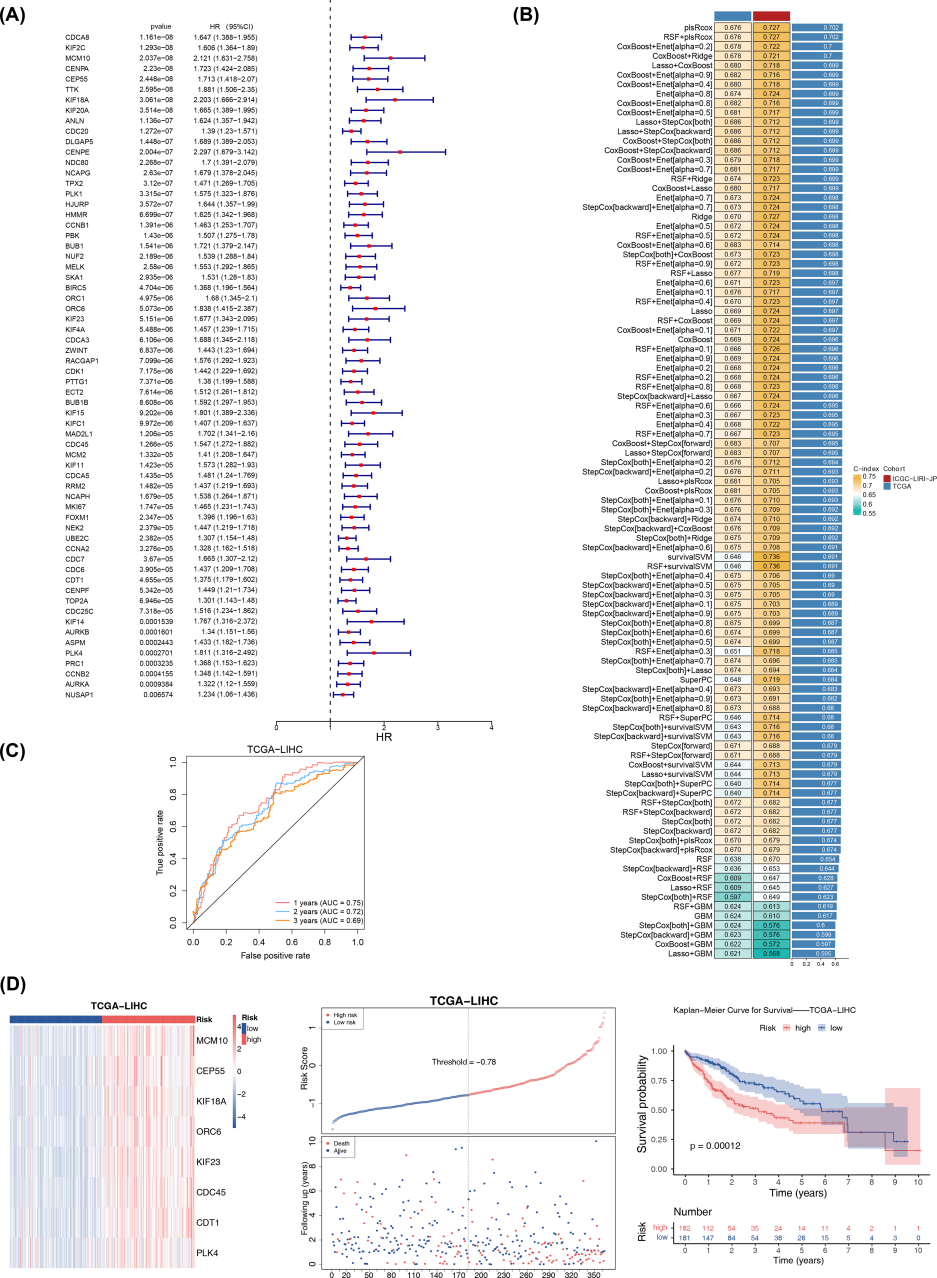
Prognostic risk model was construct in TCGA-LIHC. **(A)** Univariate Cox Forest plot of 64 genes associated with hepatocellular carcinoma. **(B)** Combined results of 101 combination algorithms. **(C)** ROC analysis illustrating the high diagnostic value of prognostic genes. **(D)** The left plot showed the heatmap of gene expression in the high- and low-risk groups based on the model (training set). The middle plot displayed the risk curve, with the x-axis representing the patient samples ordered from low to high risk based on their risk scores, with increasing risk scores from left to right. The upper part of the plot had the y-axis representing the risk scores, with red indicating high-risk group samples and blue indicating low-risk group samples. The lower part of the plot had the y-axis representing survival status, with blue indicating surviving samples and red indicating deceased samples. The dashed line represented the median risk score. The right plot displayed the Kaplan-Meier curve for the high-risk and low-risk groups based on the risk model (with the median risk score of -0.78). The x-axis represented time, the upper part of the y-axis represented survival rate, and the lower part of the y-axis represented different groups, with numbers indicating the number of surviving samples.

In the TCGA-LIHC dataset, 64 genes associated with survival were identified, and 8 of these genes were selected as candidate prognostic genes through the PH assumption test (**Figure 2A**; **Table 1**. Further screening was conducted to identify prognostic genes with prognostic value, and a risk model was constructed. In the TCGA-LIHC and ICGC-LIRI-JP, a sum of 101 combination models were constructed and the C-index for each model was calculated (**Figure 2B**). After that, the plsRcox algorithm was chosen to build risk model with C-index of 0.727, identifying MCM10, CEP55, KIF18A, ORC6, KIF23, CDC45, CDT1, and PLK4 as prognostic genes.

**Table 1:** Results of the Proportional Hazards (PH) Assumption Test.

gene	p
MCM10	0.1096
CEP55	0.3048
KIF18A	0.0772
ORC6	0.0715
KIF23	0.0616
CDC45	0.0682
CDT1	0.0981
PLK4	0.1843

The specific formula for the risk model was: RiskScore = (164.34375) × MCM10 + (117.71791) × CEP55 + (172.70708) × KIF18AC + (133.02881) × ORC6 + (113.06957) × KIF23 + (95.40480) × CDC45 + (69.54252) × CDT1 + (-520.29050) × PLK4. Subsequent ROC analysis demonstrated the high diagnostic value of these prognostic genes (AUC values > 0.6) (**Figure 2C**). Then, the HCC samples in TCGA-LIHC were categorized into high- (n = 182) and low-risk (n = 181) groups (median risk score = -0.78), where the high-risk group was significantly less likely to survive (p< 0.001) (**Figure 2D**). The risk model evaluation results obtained from two risk groups by the median risk score (median risk score = -0.74) were consistent in ICGC-LIRI-JP with those obtained in TCGA-LIHC (**Supplementary Figure S1**). Furthermore, the ROC curves and C-index results showed that the model constructed in this study performed better, and K-M survival curves showed that the risk models of Sun et al. and Zheng et al. obtained consistent and significant results with our study (p< 0.05) (**Supplementary Figure S2**). These results underscored the model’s reliability and its potential utility in prognostic assessment for HCC patients.

### 3.3 Relationship of risk model with clinical characteristics

**Figure 3:**
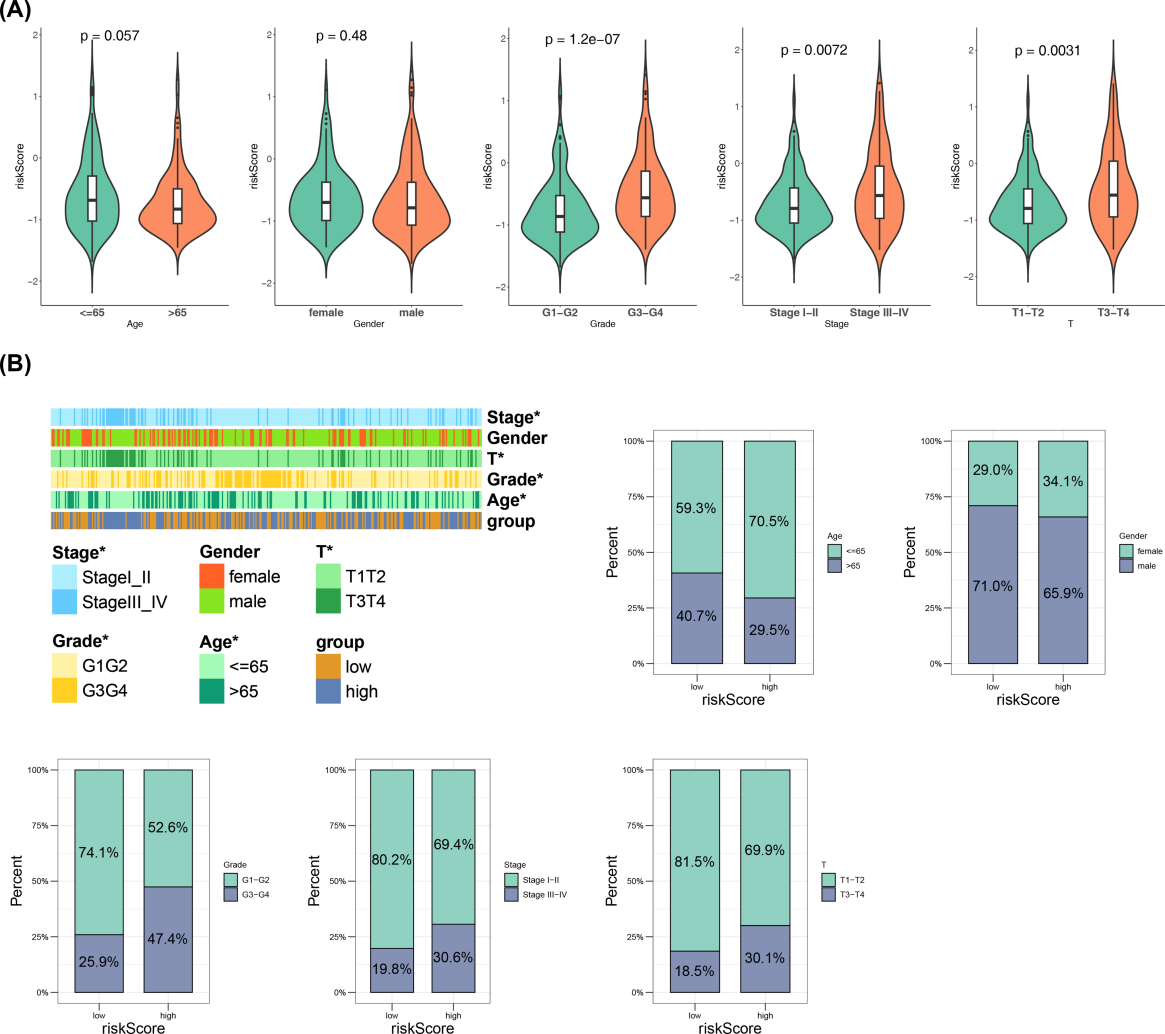
Relationship of risk model with clinical characteristics. **(A)** Violin plots of clinical feature distributions. **(B)** Heatmap of different subtypes of clinical features between high and low risk groups and distribution of risk scores in subgroups defined by clinical characteristics.

Additionally, detailed comparisons of clinical characteristics were conducted between two risk groups, including age, gender, T, stage, and grade. The violin plots showed significant differences in risk scores among T, stage, and grade subgroups **Figure 3A**). Afterwards, the percentage of males in the high-risk group was lower than that in the low-risk group, while the proportion of males was higher than that of females in both the high- and low-risk groups (**Figure 3B**; **Supplementary Table S6**), highlighting the importance of these clinical characteristics in the progression of HCC.

### 3.4 Signaling pathways analysis

**Figure 4:**
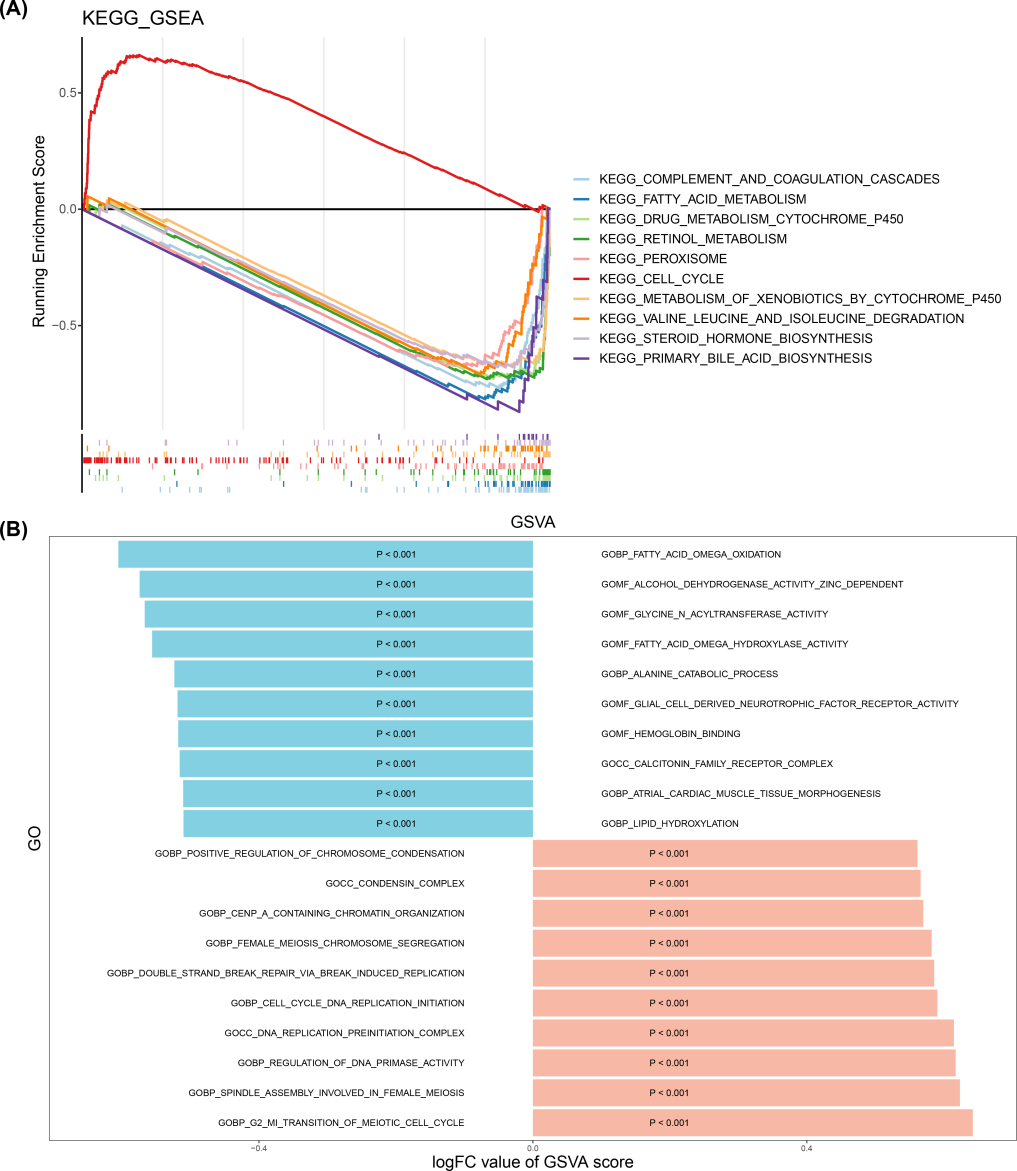
Signaling pathways analysis. **(A)** Top 10 KEGG pathway enrichment analysis results. **(B)** GSVA enrichment analysis outcomes between high- and low-risk groups (pink: represents upregulated pathways; blue: represents downregulated pathways).

To explore the signaling pathways for high- and low-risk groups, GSEA showed that a sum of 46 KEGG pathways was enriched and mainly enriched in complement and coagulation cascades (**Figure 4A**; **Supplementary Table S7**).

To further understand the differential activation pathways in two risk groups, GSVA enrichment analysis demonstrated that a total of 39 GO pathways were enriched (**Figure 4B**; **Supplementary Table S8**). Notably, the two risk groups related up-regulated GO pathways were mainly enriched in G2 MI transition of meiotic cell cycle, and the related down-regulated GO pathways were mainly enriched in fatty acid omega oxidation (p< 0.001).

### 3.5 Analysis of prognostic genes with differential immunity cells

**Figure 5:**
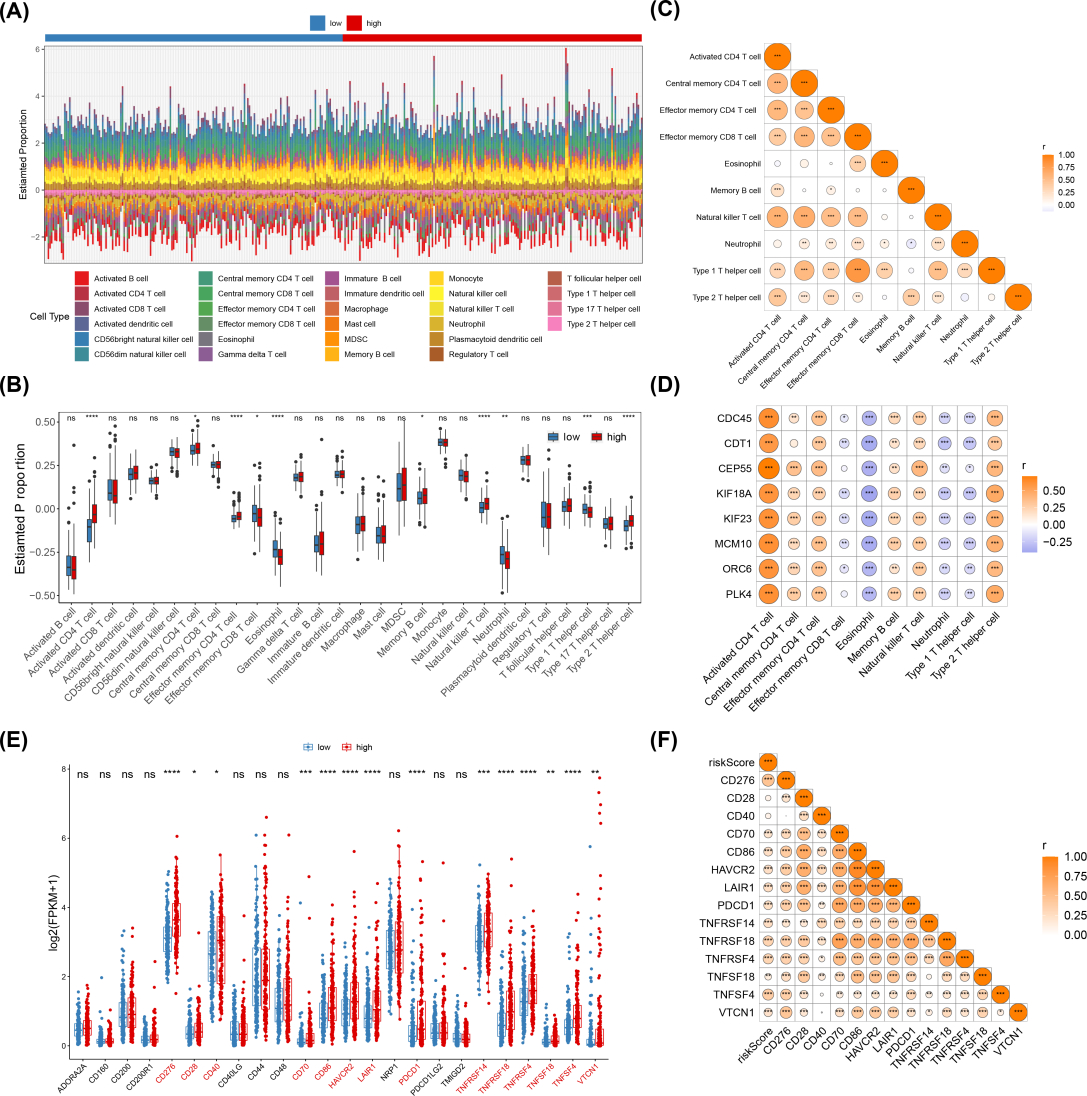
Analysis of immune cells in high and low risk groups for HCC. **(A)** Heatmap of the immune cell scores of the high-risk and low-risk groups for hepatocellular carcinoma, visualizing the difference in immune cell status between the two groups. **(B)** Box line plot of immune cell score between high risk group and low risk group. **(C)** Heat map of prognostic genes and differential immune cell correlation. **(D)** Heatmap of prognostic genes correlating with differential immune cells. **(E)** Immune checkpoint gene expression in high-risk and low-risk groups. ns: Not significant, *p< 0.05, **p< 0.01, ***p< 0.001.****p< 0.0001. **(F)** Risk score and immune checkpoint molecular correlation analysis. ns, p > 0.05; *p< 0.05; **p< 0.01; ***p< 0.001.

The objectives of this study were as follow to gain insight into the potential changes in the immune microenvironment in individuals with HCC (**Figure 5A**). Based on the results of Wilcoxon test, a sum of 10 differential immunity cells were obtained (p< 0.05), such as activated CD4 T cell (**Figure 5B**). In addition, the correlation analysis showed that type 1 T helper cell had the highest positive correlation with effector memory CD8 T cell (cor = 0.81, p< 0.001) (**Figure 5C**; **Supplementary Table S9**). Similarly, the CEP55 had the highest positive correlation with activated CD4 T cell (cor = 0.71) (p< 0.001) (**Figure 5D** and **Supplementary Table S10**). After that, a sum of 14 immune checkpoints had differences expression between two risk groups (p< 0.05) (**Figure 5E**). Following this, Spearman correlation analysis showed that risk score had the highest positive correlation with CD276 (cor = 0.46, p< 0.001) (**Figure 5F**; **Supplementary Table S11**).

### 3.6 Mutated landscapes, ESTIMATE, and drug sensitivity analysis

**Figure 6:**
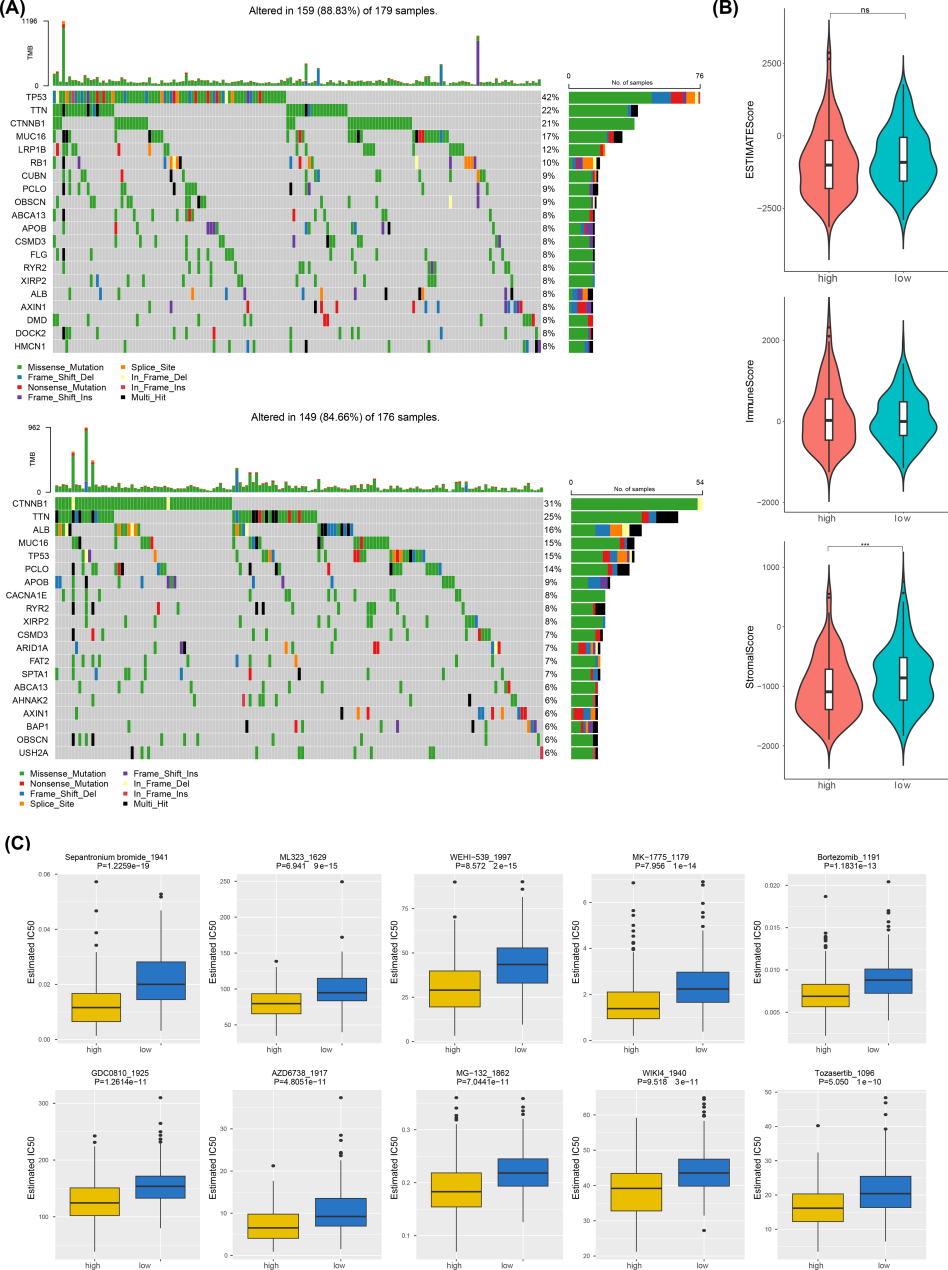
Mutated landscapes, ESTIMATE, and drug sensitivity analysis. **(A)** Shows the top 20 most frequently mutated genes in the low-risk group (below) and the high-risk group (above). **(B)** Comparison of immune score, stromal score, and composite score between high-risk and low-risk groups by ESTIMATE analysis. **(C)** Drug sensitivity analysis between high-risk and low-risk groups, with the inhibitory effect of the drug expressed as the IC50 value (50% inhibitory concentration).

The mutated landscapes were utilized to explore the function of somatic cell mutations on tumors. The waterfall plot showed that the frequency of CTNNB1 gene mutations (31%) was highest in the low-risk group, while TP53 gene mutation (42%) was highest in high-risk group and the mutation type of CTNNB1 and TP53 were both missense mutation (**Figure 6A**). Notably, only StromalScore showed significant difference between two risk groups (p< 0.001) and highly in the low-risk group (**Figure 6B**). Additionally, the drug sensitivity results indicated significant differences in IC_50_ values for 62 drugs between two risk groups in TCGA-LIHC (p< 0.05). Notably, the top 10 drugs in box plots showed that they were all highly in low- risk group (p< 0.001) (**Figure 6C**).

### 3.7 Building regulatory networks based on prognostic genes

**Figure 7:**
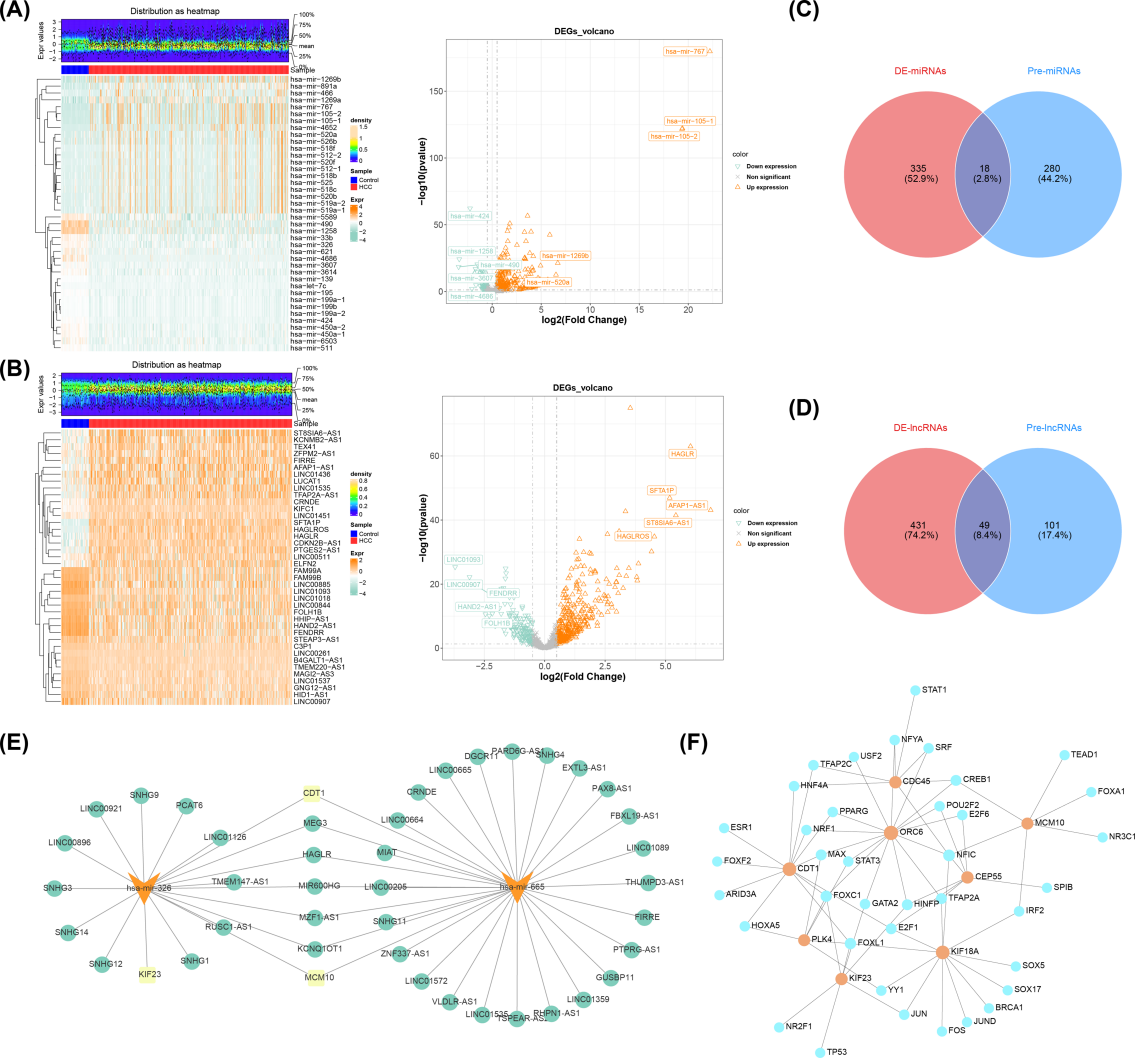
Analysis of differentially expressed miRNAs and lncRNAs in HCC and associated networks. **(A)** Heat map (left) and volcano map (right) of differentially expressed miRNAs. **(B)** Heat map (left) and volcano plot (right) of differentially expressed lncRNAs. **(C)** Venn diagram of miRNAs. **(D)** Venn diagram of lncRNAs. **(E)** Prognostic gene-miRNA-incRNA interaction network (yellow indicates prognostic genes, orange indicates miRNAs, and green indicates lncRNAs). **(F)** Prognostic gene-transcription factor (TF) regulatory network JASPAR prediction results (orange indicates prognostic genes, and blue indicates transcription factors).

A total of 353 DE-miRNAs were obtained in TCGA-LIHC (n_up_ = 288, n_down_ = 65 in HCC group) (**Figure 7A**), so as a sum of 480 DE-lncRNAs were obtained (n_up_ = 339, n_down_ = 141 in HCC group) (**Figure 7B**) (**Supplementary Table S12**). Subsequently, the starbase database identified 298 miRNAs based on MCM10, CEP55, KIF18A, ORC6, KIF23, CDT1, and PLK4. After intersecting with DE-miRNAs, a sum of 18 miRNAs were obtained in Venn diagram (**Figure 7C**), among them, the hsa-mir-326 and hsa-mir-665 were down regulated miRNAs in HCC group (MCM10, CEP55, KIF18A, ORC6, KIF23, CDT1, and PLK4 were up regulated mRNAs in HCC group) (**Supplementary Table S13**), thus hsa-mir-326 and hsa-mir-665 were regarded as key miRNAs. Similarly, a total of 150 lncRNAs were predicted by 2 key miRNAs, then 49 lncRNAs were obtained in Venn diagram (**Figure 7D**), and 40 up regulated lncRNAs in HCC group were finally obtained called key lncRNAs (**Supplementary Table S14**). Lastly, in the key lncRNAs-key miRNAs-prognostic genes regulatory network, the CDT1 and MCM10 both predicted 2 key miRNAs, furthermore, hsa-mir-326 and hsa-mir-665 both predicted 5 key lncRNAs, which were MEG3, HAGLR, MIR600HG, MZF1-AS1, and KCNQ1OT1 (**Figure 7E**). Furthermore, the JASPAR prediction results from the Networkanalyst platform indicated that a grand total of 35 TFs were predicted by 8 prognostic genes, the CDC45, ORC6, and MCM10 all predicted CREB1 (**Figure 7F**).

### 3.8 A comprehensive single-cell sequencing analysis in HCC

**Figure 8:**
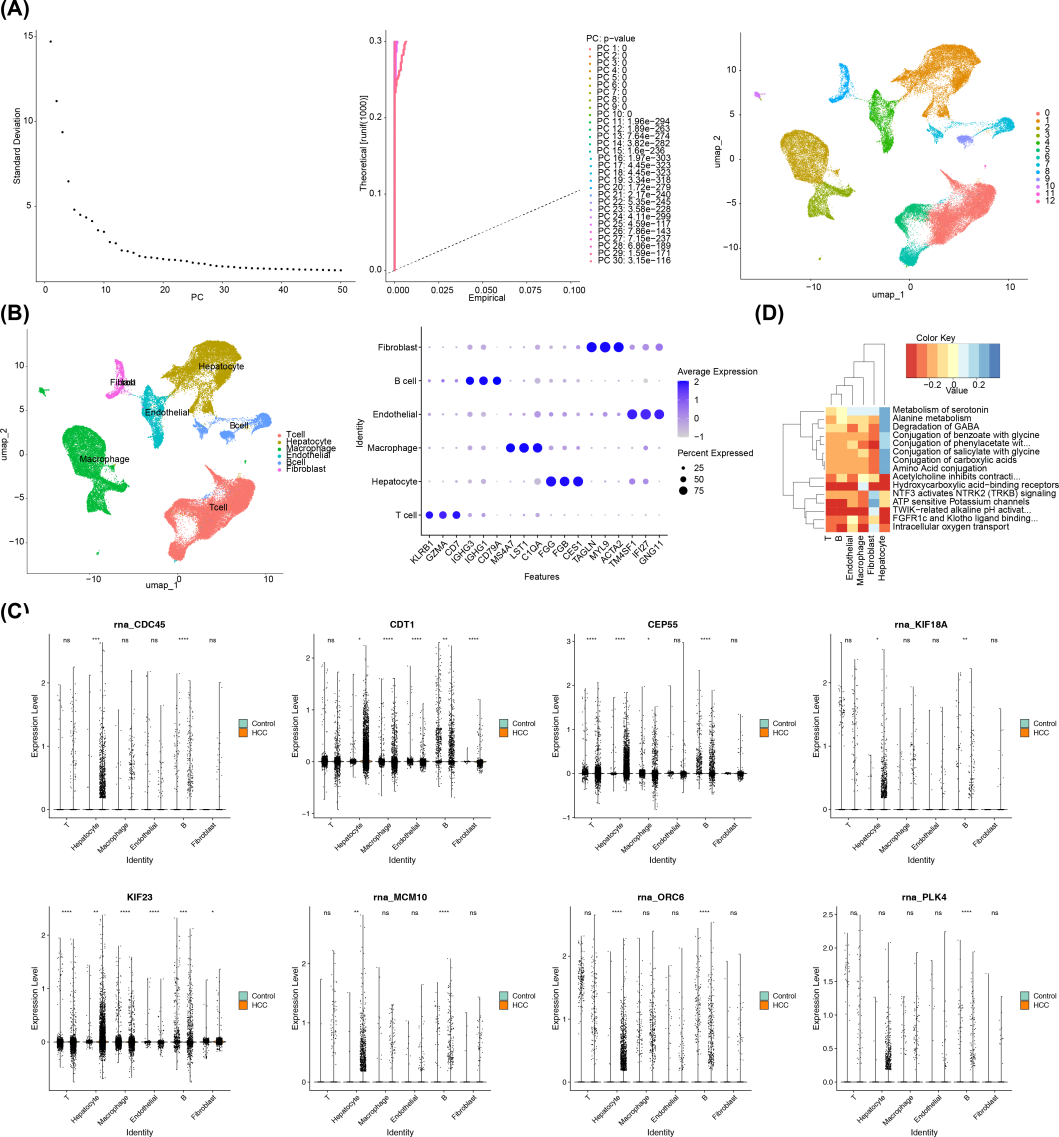
A comprehensive single-cell sequencing analysis in HCC. **(A)** PCA displacement test and inflection plot; cell UMAP clustering plot. **(B)** The left panel showed the cell clustering subpopulation annotation results, and the right panel showed the gene bubble map for each cluster annotation marker. **(C)** Differential expression of prognostic genes in subpopulations. The left column is the control group and the right column is the HCC group. **(D)** Enrichment pathway of key cells. The Color Key represented the scores of the gene set in the cells, indicating the overall expression level of the gene set in the cells. Red indicated that the gene set was overall lowly expressed in the cells, while blue indicated that the gene set was overall highly expressed in the cells.

Following the initial screening, a sum of 57,741 cells and 24,863 genes were obtained on the basis of the GSE149614. After normalizing and downscaling the data, 2,000 highly variable genes, 30 PCs and 13 cell clusters were obtained (**Figure 8A**). A whole of 13 types of cell populations were annotated to 6 cell types, including T cells, hepatocytes, macrophages, endothelial cells, B cells, and fibroblasts (**Figure 8B**). In addition, the levels of expression of the marker genes in the corresponding cell types had also been demonstrated in this study (**Supplementary Figure S3**).

Afterwards, the 8 prognostic genes all had significant differences between HCC and control groups in B cells, thus the B cells were regarded as key cells (p< 0.05) (**Figure 8C**). Afterwards, according to the heat map, the pathways of B cells were mainly enriched in intracellular oxygen transport, TWIK-related alkaline pH activation, adenosine triphosphate (ATP) sensitive potassium channels, and hydroxycarboxylic acid−binding receptors (**Figure 8D**).

### 3.9 Cell communication and cell trajectory analysis of B cells

**Figure 9:**
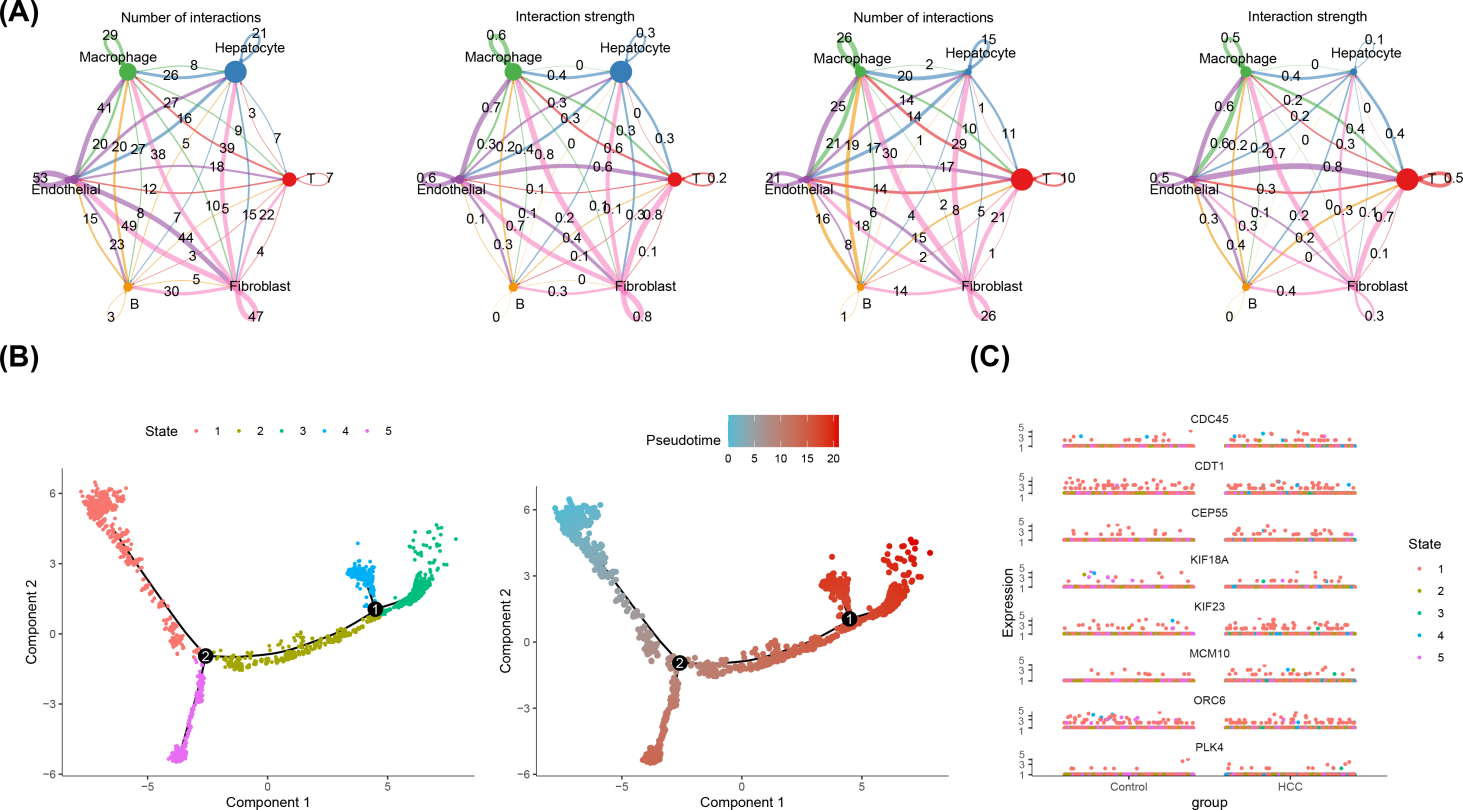
Cellular Communication and Proposed Timing Analysis. **(A)** Cellular communication network of key cells in HCC group (left two figures) and control group (right two figures). ‘Number of Interactions’ represents the frequency of cell-to-cell interactions; ‘Interaction Strength’ indicates the strength of cell-to-cell interactions. **(B)** Proposed time trajectory analysis of key B cells. The left panel shows the proposed time trajectory, with the transition from blue to red indicating the order of cell differentiation. The right panel shows the various states of the cell throughout the differentiation process. **(C)** Differential expression of 8 prognostic genes over time in the control and HCC groups.

Additionally, cell communication analysis revealed interactions between B cells and other cell types. The analysis of cell communication revealed that the B cells had highest interaction number and interaction strength with macrophages (**Figure 9A**). Among them, in HCC group, fibroblasts and T cells had highest interaction weight, while in control group, endothelial cells and T cells had highest interaction weight (**Supplementary Table S15**). Furthermore, this study also showed the results of ligand-receptor pairing among 6 cell types. The results demonstrated that there were a greater number of receptor-ligand interactions between cells in the HCC group (**Supplementary Figure S4**). In particular, both in HCC and control groups, the most pronounced effect was observed in the APP-CD74 interaction between endothelial-B cell (p< 0.01) (**Supplementary Table S16**). This study demonstrated that the B cell differentiation in 5 States, the State 1 was the beginning of differentiation, and the State 4 and 5 were the two states of the ending differentiation (**Figure 9B**). The intensity of the color in the graph was indicative of the timing of cell differentiation, with darker shades representing earlier periods. Notably, according to the results of the distribution in 8 prognostic genes, it was known that prognostic genes were more highly expressed in State 1, the beginning of B cell differentiation (**Figure 9C**).

### 3.10 Expression validation analysis

**Figure 10:**
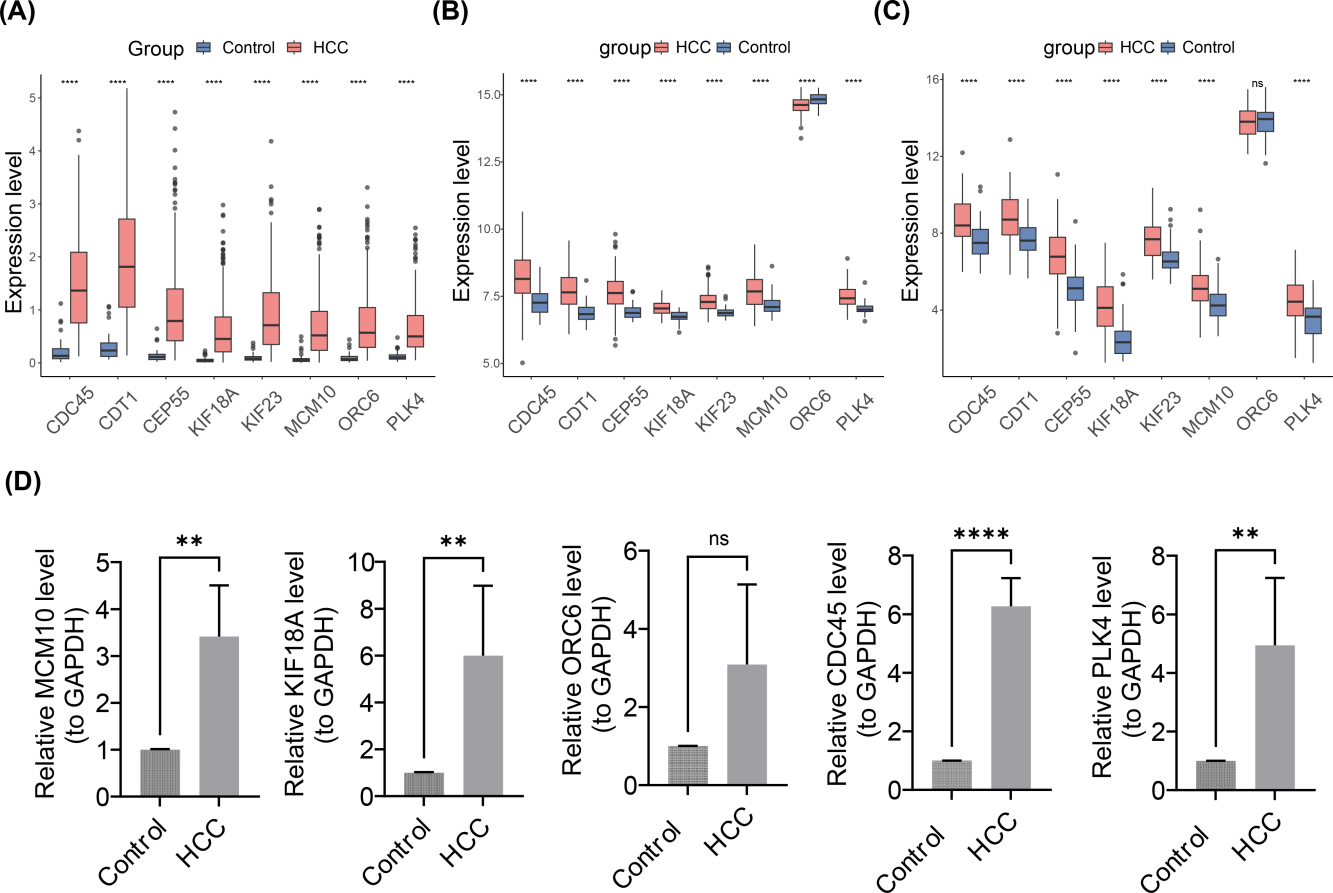
Expression Validation Analysis of Prognostic Gene in Control and HCC Groups. **(A-C)** Box plots of the expression profiles of 8 prognostic genes. **(A)** TCGA-LIHC dateset. **(B)** GSE76427 dataset. **(C)** GSE54236 dataset. ns represented no significance, **** represented p< 0.0001. **(D)** Reverse transcription quantitative polymerase chain reaction expression validation. From left to right are MCM10, KIF18, ORC6, CDC45, PLK4. ns represented not significant, ** represented p< 0.01, **** represented p< 0.0001.

Notably, in the HCC group, the box plots showed that the expression of 8 prognostic genes was significantly upregulated (p< 0.001) (**Figure 10A**). Meanwhile, in the GSE76427 and GSE54236 datasets, except for ORC6, the expression levels of the other seven genes were significantly higher in the disease group compared to the control group (**Figures 10B, C**). Expression validation analysis revealed differences in prognostic genes (MCM10, KIF18A, ORC6, CDC45, and PLK4) in control and HCC groups. The results showed that the expression of MCM10, KIF18A, ORC6, CDC45, and PLK4 in HCC group notably elevated compared to the control group (**Figure 10D**). It was worth noting that, MCM10, KIF18A, CDC45, and PLK4 showed significant differences between two groups (p< 0.05). Based on the results of expression, it was found that the expression trends of MCM10, KIF18A, ORC6, CDC45, and PLK4 were consistent with the results of the Wilcoxon test, which provided a reference for subsequent studies.

## 4 Discussion

HCC frequently presents with nonspecific symptoms, resulting in limited opportunities for curative surgery and a five-year survival rate of approximately 20% (42). Given this context, there is an urgent need for research aimed at improving HCC prognosis. A total of eight prognostic genes for HCC (MCM10, CEP55, KIF18A, ORC6, KIF23, CDC45, CDT1, and PLK4) were identified by analyzing data from the UCSC Xena, ICGC, and GEO databases. Based on these genes, a reliable prognostic model for HCC was constructed, offering significant clinical implications for patient prognosis and treatment strategies. Furthermore, at the single-cell level, our study elucidated the molecular characteristics of distinct cell populations within HCC and identified key cell types, providing a theoretical foundation for further exploration of their potential immune mechanisms.

In this study, the eight prognostic genes were intricately linked to the prognosis of HCC, which were selected through a rigorous process involving univariate Cox regression analysis, the PH assumption test, and 101 machine learning algorithms. The model was constructed utilizing these genes exhibits superior predictive accuracy compared to contemporaneous predictive models, thereby offering a reliable tool for clinical practice.

MCM10 has been highlighted for its significant overexpression in various cancer tissues and its association with tumor aggressiveness, immune cell infiltration, immune checkpoints, tumor mutational burden (TMB), and microsatellite instability (MSI) (43). Its potential as a therapeutic target and prognostic biomarker has been substantiated in endometrial carcinoma (44), with similar trends observed in HCC patients (45). CEP55, a centrosomal protein, influences cell mitosis and has been implicated in the progression of multiple malignancies. Its elevated expression in tumors compared to normal tissues correlates with patient pathological grading and age, making it a promising target for therapeutic intervention in HCC (46, 47). CDT1 is a key regulator in the cell cycle and DNA replication, and has been shown to promote the occurrence and development of liver cancer (48, 49). KIF18A and KIF23 are members of the kinesin family and were overexpressed in many malignant tumors. Ren et al (50) had proved KIF18A could mediate proliferation and metastasis in HCC cells. Also, KIF23 had been reported to correlate with the cell proliferation, invasion, and migration of the HCC cells (51), and as a potential biomarker to predict the poor prognosis of HCC (52). ORC6 is the smallest ORC subunit, which is essential in DNA replication initiation (53). Similarly, ORC6 could promote the proliferation, migration, invasion of HCC cells (54). CDC45 encodes the protein to regulate the initiation and elongation stages of eukaryotic chromosomal DNA replication (55), which was overexpressed in HCC and correlated with worse prognosis (56). The last identified gene PLK4 is a serine/threonine kinase and it causes centrosome amplification, aneuploidy, and genomic instability. Yeung et al. (57) discovered that PLK4 plays an important role in HCC metastasis. RT-qPCR validation confirmed significant differences in MCM10, KIF18A, CDC45, and PLK4 expression between HCC and control groups. These genes are closely associated with HCC prognosis, thereby corroborating the results of our previous bioinformatics analysis. Additionally, some predicted genes showed opposite or non-significant trends in PCR validation, which may be due to small sample size or inconsistent sample sources. The role of these genes in disease progression will continue to be monitored.

The predictive model based on these prognostic genes, demonstrates a higher predictive effect compared to other models, suggesting its potential for broad clinical application (23–25). This model not only aids in the prognostication of HCC patients but also informs treatment strategies, emphasizing the importance of these genes in the prognosis and management of HCC.

In this study, gender-based risk stratification of HCC patients revealed a higher proportion of males in the low-risk subgroup compared to females, a phenomenon potentially attributable to estrogen-mediated protective effects in females. Mechanistically, estrogen has been demonstrated to maintain cholesterol homeostasis through LCAT induction and suppress hepatocarcinogenesis (58). Notably, the survival advantage conferred by estrogen in females appears particularly pronounced during the perimenopausal period (45-59 years), likely associated with dynamic fluctuations in estrogen levels (59). Our findings showing a higher male proportion in the low-risk group (71% vs. 65.9% in high-risk group) may reflect increased representation of perimenopausal females in the low-risk cohort. Pathophysiological evidence further indicates altered sex hormone receptor profiles in HCC tissues, characterized by upregulated androgen receptor (AR) expression and diminished estrogen receptor (ER) levels (60) potentially compromising estrogen’s protective capacity. This attenuation appears exacerbated in advanced-stage patients or those with severe cirrhosis, where hepatic estrogen metabolism may be substantially impaired (61). Intriguingly, the overrepresentation of advanced-stage cases in our high-risk cohort might partially obscure estrogen-mediated protection, resulting in a relatively lower male proportion compared to the low-risk group.

Although the proportion of males in the high-risk group is lower than that in the low-risk group, the overall risk remains higher for males in the high-risk group. This suggests that males, even when categorized in the low-risk group, should still be vigilant regarding the potential risk of liver cancer. Therefore, gender differences, the protective effects of estrogen, and the unique physiological state of perimenopausal women may all influence gender distribution and disease progression across different risk groups.

Through GSEA analysis, the study identified 46 KEGG pathways, with these pathways providing significant insights into HCC. Notably, the complement and coagulation cascades pathway underscores the liver’s integral role in synthesizing over 80% of complement components and expressing various complement receptors, forming an innate defense system known as the complement cascade (CC). This system tightly regulates humoral and cellular responses to harmful stimuli, with the complement cascade activating immune cells critical to HCC pathogenesis (62). Additionally, fatty acid metabolism and retinol metabolism were identified as key pathways in HCC, necessitating further exploration for the development of novel therapeutic strategies (63). The valine, leucine, and isoleucine degradation pathway, confirmed as highly enriched in 2018, not only elucidates the pathogenesis of HCC but also provides prognostic markers and therapeutic targets (64). Bile acid metabolism plays a pivotal role in modulating the tumor immune microenvironment, with the primary bile acid biosynthesis pathway correlating with HCC prognosis (65, 66). Human cytochrome P450 (CYP), a bidirectional membrane protein, encompasses 18 families with a total of 57 functional genes, participating in drug metabolism and the homeostasis of fatty acids, vitamin D, steroids, and bile acids, as well as pathological physiological processes in certain cancers or cardiovascular diseases, presenting substantial therapeutic potential (67). The drug metabolism cytochrome P450 pathway was found enriched in the SMYD5 high expression phenotype, suggesting it as a potential biomarker for HCC prognosis and treatment (68). CYPs have a dynamic role in HCC pathogenesis, and down-regulated CYPs could increase susceptibility to drug toxicity (69, 70). These pathways, through various mechanisms, influence HCC tumor cell differentiation and proliferation, promoting HCC progression and emerging as potential therapeutic targets.

This study also reveals significant correlations between the immune microenvironment in HCC and prognostic genes. Particularly, 10 differentially expressed immune cells, such as activated CD4 T cells, natural killer T cells, and Type 1/2 T helper cells, show notable associations with prognostic genes. Notably, Type 1 T helper cells exhibit the highest positive correlation with effector memory CD8 T cells, while CEP55 demonstrates the strongest positive correlation with activated CD4 T cells. Elevated expression of Type 1 T helper cells is known to increase tumor cells’ sensitivity to various treatments, and when combined with clinical parameters like TNM staging and AFP levels, it presents excellent predictability of early HCC recurrence (71). Activated CD4 T cells can regulate immune surveillance in HCC, thereby inhibiting the development of liver cancer (72). In the tumor-promoting NAFLD liver microenvironment, NKT cell dysfunction occurs, hence, invigorating NKT cells could control HCC in the obesity epidemic (73). Previous studies have shown that lower peripheral blood eosinophil counts are associated with poorer prognosis (74), and elevated neutrophil extracellular traps facilitate the growth and metastasis of HCC (75). Furthermore, a high neutrophil to eosinophil ratio may be correlated with a higher recurrence of HCC (76). In this study, the aforementioned immune cells were found to infiltrate less in both high- and low-risk HCC groups, suggesting that immune suppression in HCC may be quite prevalent, and there might be a deficiency in the normal immune surveillance within the tumor microenvironment.

Furthermore, Spearman correlation analysis indicates that the risk score has the highest positive correlation with CD276, also known as B7-H3, a type I transmembrane protein belonging to the B7 family of immune regulatory molecules. Acting as an immune checkpoint molecule, CD276 plays a crucial role in immune evasion and the tumor immune microenvironment (77). Overexpression of CD276 promotes an inhibitory tumor microenvironment in HCC and is associated with poor prognosis (78). This suggests that the risk score is implicated in tumor progression and the prediction of patient outcomes. TMB reflects the degree of genomic variation in tumor cells. In HCC, a high TMB is associated with reduced survival rates and predicts a better response to immunotherapy (79). ESTIMATE is a method that utilizes cancer transcriptome expression data to estimate the content of stromal cells and immune cells in malignant tumor tissues. Studies have shown that in patients with liver cancer, the expression of miR-148a-3p is significantly negatively correlated with the stromal score, immune score, and ESTIMATE score, and low expression of miR-148a-3p can serve as a prognostic and diagnostic marker for HCC (80). These findings underscore the importance of the immune microenvironment in the prognosis of HCC and highlight the potential of immune-related genes as predictive biomarkers and therapeutic targets.

This study explores the therapeutic potential of the top 10 ranked drugs for HCC, highlighting significant advancements in drug research. Pharmacological screening has identified Sepantronium Bromide as a promising antitumor agent, particularly effective in lenvatinib-resistant HCC patients (81). Additionally, ML-323 has been demonstrated to inhibit HCC cell growth and induce G1 phase cell cycle arrest by regulating cyclin expression (82). In combination with BPD-00008900, Doramapimod, and AZD2014, these chemical compounds have shown enhanced chemotherapy outcomes for high-risk patients within specific subgroups (83). Furthermore, Bortezomib, acting through the Hippo-YAP signaling pathway, emerges as an effective anti-HCC drug (84). The drugs discussed in this study have all been shown to play a significant role in the development and progression of tumors, including HCC. Therefore, this research provides substantial reference value for the future investigation of these drugs in the treatment of HCC.

This study revealed that B cells are considered key cells for the 8 prognostic genes. B cells have the highest number and strength of interactions with macrophages and are differentiated into 5 states. State 1 marks the beginning of B cell differentiation, while States 4 and 5 represent the end stages of differentiation. Notably, the prognostic genes are more highly expressed in State 1, the initial stage of B cell differentiation. B cells are recognized as the primary effector cells of humoral immunity, capable of inhibiting tumor progression, and their role in the TME is of great interest. The presence of B cells has been associated with improved outcomes in cancer patients (85, 86). In HCC, IgG+ plasma cells can be recruited by tumor-associated macrophages (TAMs) via the CXCR3-CXCL10 axis, promoting the formation of protumor macrophages and thereby enhancing immune suppression (87). Meanwhile, B cells can capture tumor-associated antigens via the B cell receptor (BCR), and after internalization and processing, present them to CD4+ and CD8+ T cells to exert antitumor immune effects (87). Therefore, the pivotal role of B cells in HCC prognosis requires further research, providing a scientific basis for therapeutic strategies targeting these cells.

In summary, this study identified eight prognostic genes relevant to HCC—MCM10, CEP55, KIF18A, ORC6, KIF23, CDC45, CDT1, and PLK4—and constructed a validated HCC prognostic risk prediction model. Furthermore, B cells were identified as key cellular components associated with these prognostic genes. The overexpression of MCM10, KIF18A, CDC45, and PLK4 in the HCC group was confirmed through bioinformatics analysis and RT-qPCR, which could be particularly significant for identifying new therapeutic targets for HCC. However, our study has several limitations. First, the PCR results were not entirely consistent with the dataset, which may be attributed to the small sample size, sample heterogeneity, and differences in the algorithm parameters of bioinformatics analysis as well as the reaction conditions of the RT-qPCR experiments. Second, the lack of P-value correction during the selection of differentially expressed genes may have led to false-positive results. Additionally, the absence of in-depth functional experiments and multivariate analysis of confounding factors limits the comprehensive understanding of the true relationship between the risk score and clinical features. To address these limitations, we plan to expand the sample size and collect diverse sample types in future studies. We will also employ appropriate correction methods to minimize false positives. Furthermore, we aim to establish mouse or cell models to conduct *in vivo* and *in vitro* functional experiments. These experiments will utilize techniques such as immunohistochemistry, ELISA, flow cytometry, Western blotting, and gene editing to validate the impact of prognostic genes and key cells on HCC. Specifically, we will use CRISPR/Cas9 gene-editing technology to knock out or overexpress prognostic genes with significant expression differences in B cells, thereby observing their effects on B cell function. Future research will also incorporate more confounding factors into a comprehensive analysis to more fully assess the relationship between the risk score and clinical features.

## 5 In conclusion

This study identified eight prognostic genes—MCM10, CEP55, KIF18A, ORC6, KIF23, CDC45, CDT1, and PLK4—and developed a robust predictive model for HCC. This model offers a new direction for research and could significantly assist in the clinical management of the disease.

## Data Availability

The original contributions presented in the study are included in the article/**Supplementary Material**. Further inquiries can be directed to the corresponding author/s.
