# Supplementary material for: Clinical potential and experimental validation of prognostic genes in hepatocellular carcinoma revealed by risk modeling utilizing single cell and transcriptome constructs
Source: Front Immunol. 2025 Apr 4;16:1541252. doi: 10.3389/fimmu.2025.1541252 (PMC12006083; doi:10.3389/fimmu.2025.1541252)
Supplement: Supplementary file 1 [file DataSheet1.zip › legends for supplementary figures and tables.docx]

**Supplementary Figures:**

**Supplementary Figure 1: The risk model evaluation results of ICGC-LIRI-JP.**From top to bottom and then from left to right: The heatmap, Kaplan-meier curve, survival curves, and Receiver operating characteristic curves in ICGC-LIRI-JP by the median risk score -0.74.

**Supplementary Figure 2: The risk model evaluation results compared to other papers.**From top to bottom and then from left to right: The C-index results of ICGC-LIRI-JP, PMID 37051238, PMID 30538557, and PMID 37520528；The Kaplan-meier curve, ROC curves of PMID 37051238, PMID 30538557, and PMID 37520528.

**Supplementary Figure 3: The expression levels of the marker genes in the corresponding cell types.**From top to bottom and then from left to right: Single cell data filtering results; PCA sample cell distribution map; Screening map of hypervariable genes; Violin map of marker gene in each cell group.

**Supplementary Figure 4: The receptor-ligand interactions between cells in the control and HCC groups.**

**Supplementary Tables:**

**Supplementary Table 1 The procedure information of real-time quantitative PCR.**

**Supplementary Table 2 The primer sequences of real-time quantitative PCR.**

**Supplementary Table 3 The data sheet of GO analysis.**

**Supplementary Table 4 The data sheet of KEGG pathways analysis.**

**Supplementary Table 5 The obtained 64 candidate genes from PPI network analysis.**

**Supplementary Table 6 Table of different clinical subtypes between high-risk and low-risk groups**

**Supplementary Table 7 The data sheet of 46 KEGG pathways.**

**Supplementary Table 8 The enriched 39 GO pathways obtained from GSVA enrichment analysis.**

**Supplementary Table 9 The Spearman correlation analysis results of the 10 differential immunity cells.**

**Supplementary Table 10 The Spearman correlation analysis results of the 8 prognostic genes.**

**Supplementary Table 11 The Spearman correlation analysis results among the 8 prognostic genes and the 10 differential immunity cells.**

**Supplementary Table 12 The obtained 353 DE-miRNAs and 480 DE-lncRNAs in TCGA-LIHC.**

**Supplementary Table 13 The down regulated hsa-mir-326 and hsa-mir-665 in HCC group.**

**Supplementary Table 14 The 40 up regulated lncRNAs in HCC group.**

**Supplementary Table 15 The results of cell communication analysis.**

**Supplementary Table 16 The data sheet of the results of ligand-receptor pairing between cells in the HCC group.**
