# Supplementary material for: Clinical potential and experimental validation of prognostic genes in hepatocellular carcinoma revealed by risk modeling utilizing single cell and transcriptome constructs
Source: Front Immunol. 2025 Apr 4;16:1541252. doi: 10.3389/fimmu.2025.1541252 (PMC12006083; doi:10.3389/fimmu.2025.1541252)
Supplement: Supplementary file 1 [file DataSheet1.zip › Supplementary Table 1.docx]

|  | **Temperature** | **Time** |
| --- | --- | --- |
| Initial denaturation | 95℃ | 1min |
| Denaturation | 95℃ | 20s |
| Annealing | 55℃ | 20s |
| Txtension | 72℃ | 30s |
