# Supplementary material for: Clinical potential and experimental validation of prognostic genes in hepatocellular carcinoma revealed by risk modeling utilizing single cell and transcriptome constructs
Source: Front Immunol. 2025 Apr 4;16:1541252. doi: 10.3389/fimmu.2025.1541252 (PMC12006083; doi:10.3389/fimmu.2025.1541252)
Supplement: Supplementary file 1 [file DataSheet1.zip › Supplementary Table 2.docx]

| **Primer** | **Sequence** | |
| --- | --- | --- |
| MCM10 F | GGGGAAACGACTCAACCCAT | |
| MCM10 R | CACACTCTGTGGCGTAACCT | |
| KIF18A F | GACTGGGTGTTGAGTTAGCAGA |  |
| KIF18A R | TCTCGTTTTCCAGTGGCCTT |  |
| ORC6 F | GTCCAGTCTTCCCCAGACAC |  |
| ORC6 R | CCATTTCCTTTGCTGGGGCT |  |
| CDC45 F | TTTGGGAGGGCGTTTGAGAA |  |
| CDC45 R | AACTTGCTCCGATCCTCAGC |  |
| PLK4 F | CTTTCCGTGGTTTCAGCGTC |  |
| PLK4 R | TTCCTTCACGCAGTCCCTTC |  |
| GAPDH F | CGAAGGTGGAGTCAACGGATTT |  |
| GAPDH R | ATGGGTGGAATCATATTGGAAC |  |
