# Supplementary figures and images for: Clinical potential and experimental validation of prognostic genes in hepatocellular carcinoma revealed by risk modeling utilizing single cell and transcriptome constructs
Source: Front Immunol. 2025 Apr 4;16:1541252. doi: 10.3389/fimmu.2025.1541252 (PMC12006083; doi:10.3389/fimmu.2025.1541252)

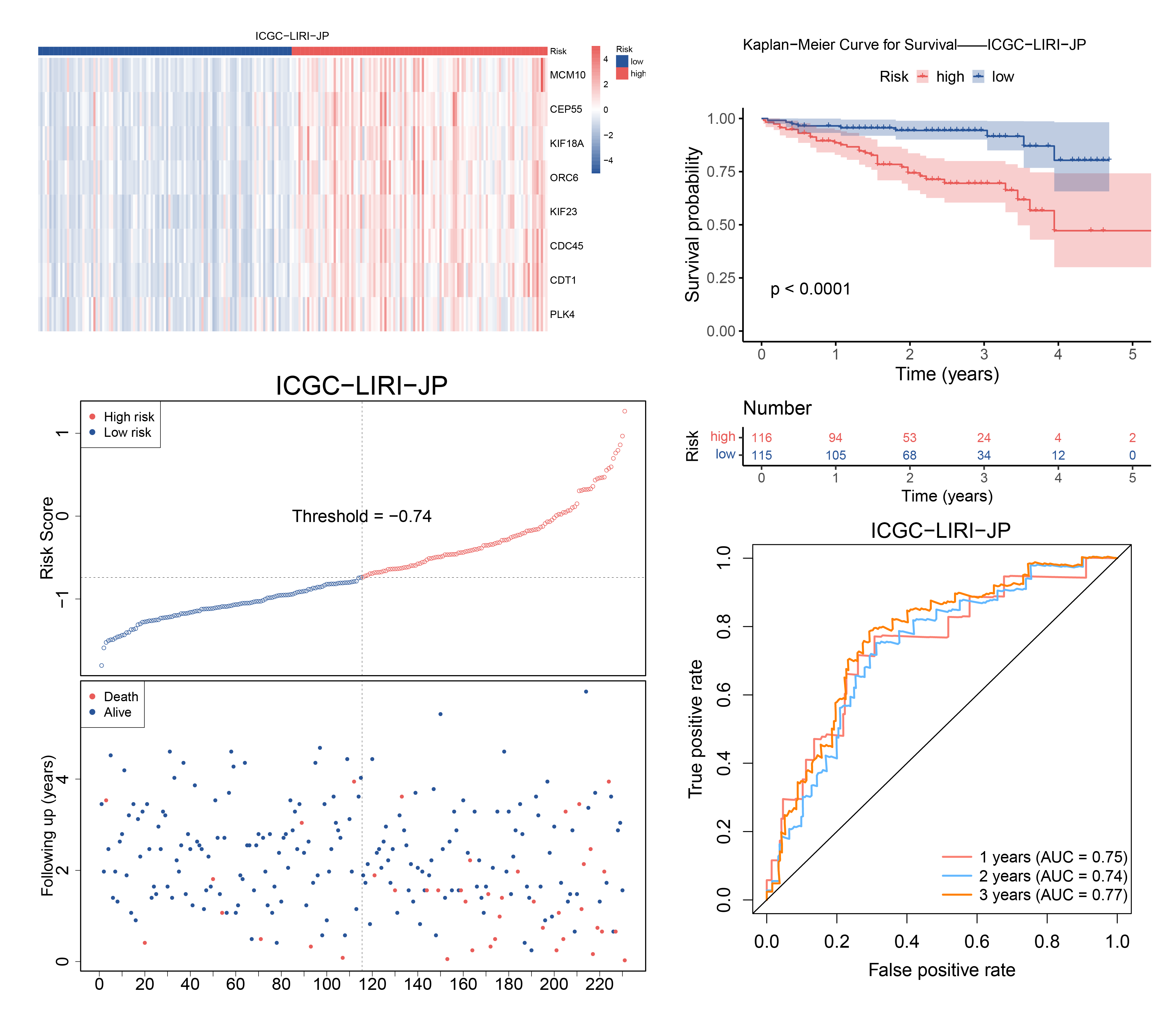

Supplement: Supplementary file 1 [file DataSheet1.zip › supplememtary Figure 1.tif]

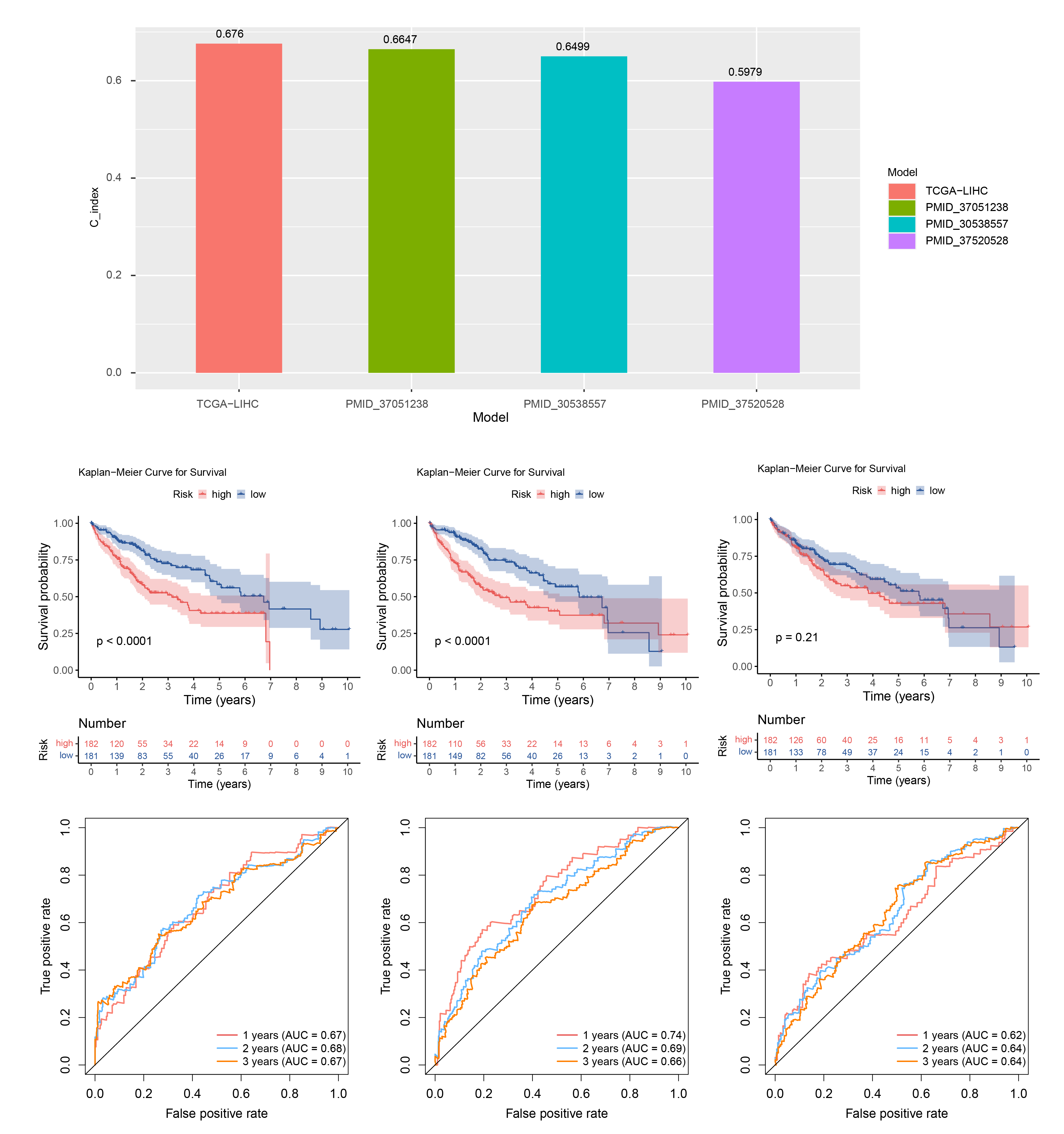

Supplement: Supplementary file 1 [file DataSheet1.zip › supplememtary Figure 2.tif]

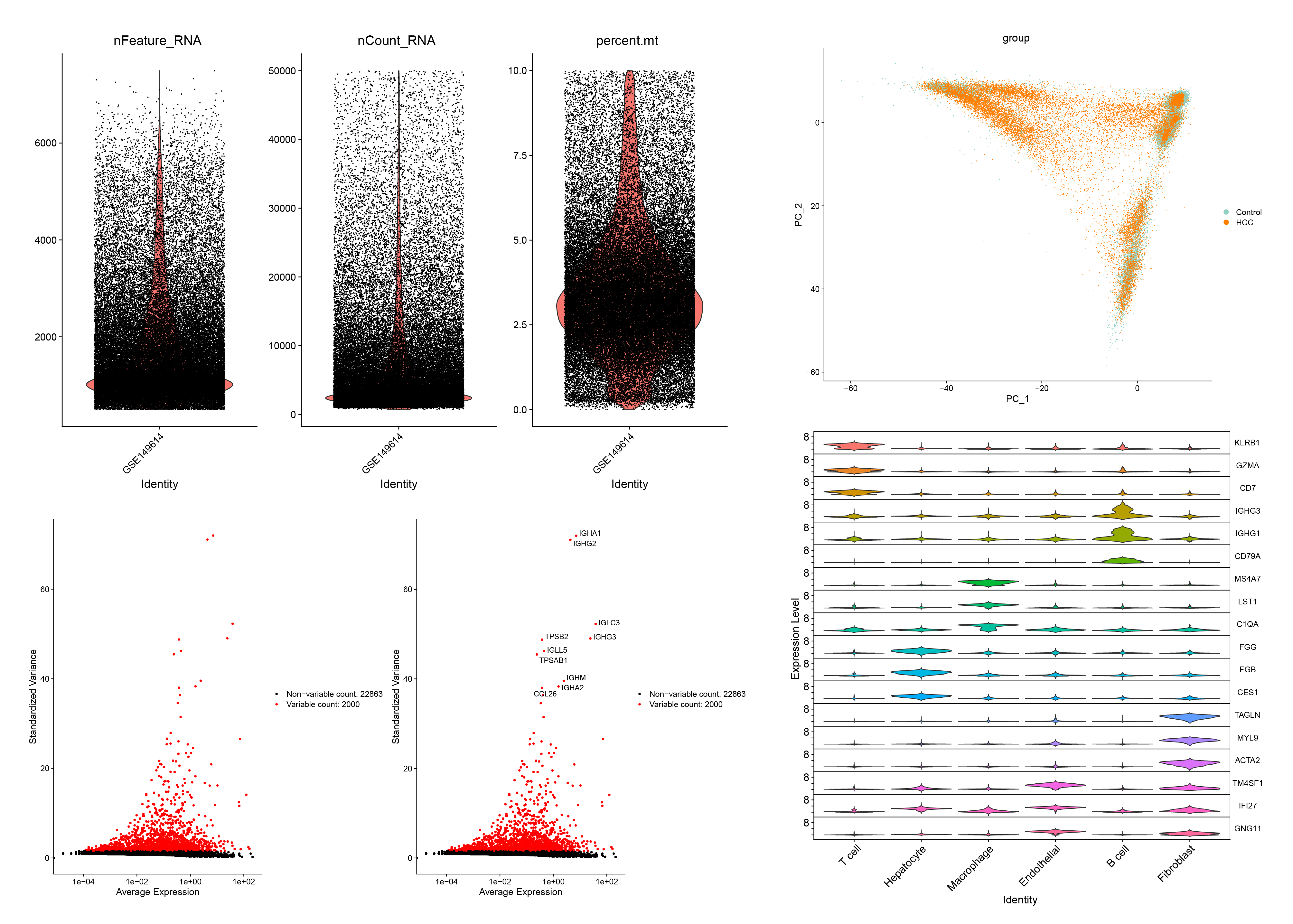

Supplement: Supplementary file 1 [file DataSheet1.zip › supplememtary Figure 3.tif]

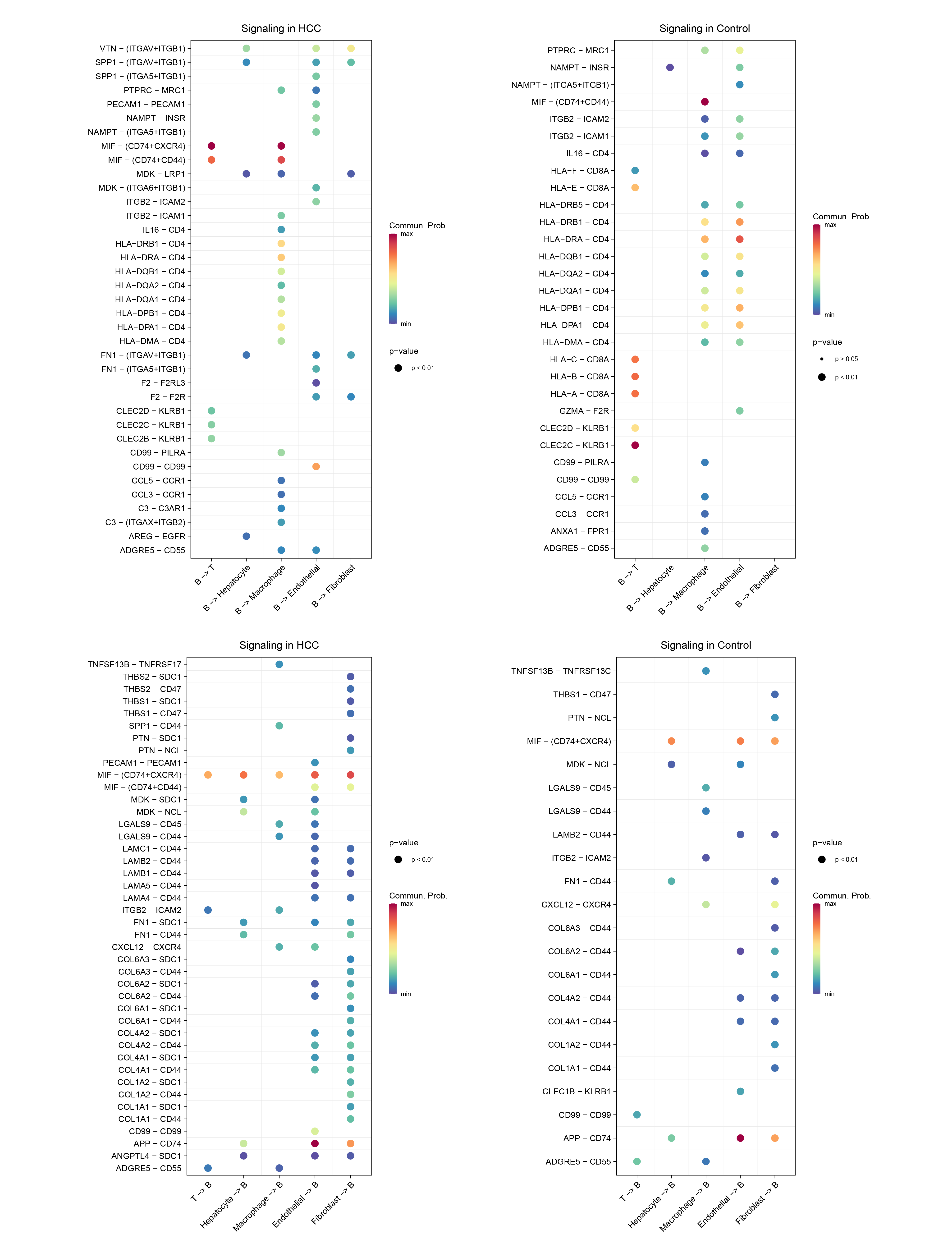

Supplement: Supplementary file 1 [file DataSheet1.zip › supplememtary Figure 4.tif]
